# Association between volatile organic compound concentrations in daycares and child wheeze: the French CRESPI study

**DOI:** 10.1186/s12940-026-01307-6

**Published:** 2026-05-13

**Authors:** Ioannis A. Sakellaris, Corinne Mandin, Franziska Bright, Laurent Orsi, Flore Amat, Pierre Bonnet, Valérie Siroux, Nicole Le Moual, Orianne Dumas

**Affiliations:** 1 Université Paris-Saclay, UVSQ, Univ. Paris-Sud, Inserm, Équipe d’Épidémiologie Respiratoire Intégrative, CESP, Villejuif, 94807 France; 2 Centre Scientifique et Technique du Bâtiment (CSTB), Direction Santé Confort, 84, avenue Jean Jaurès, Marne-la-Vallée, France; 3 Service de Pneumologie et d’Allergologie Pédiatrique - CRCM, Hôpital Robert Debré, Université Paris Cité, Paris, France; 4 Team of Environmental Epidemiology Applied to the Development and Respiratory Health, Institute for Advanced Biosciences, Inserm U 1209, CNRS UMR 5309, Université Grenoble Alpes, Grenoble, France

**Keywords:** VOCs, Aldehydes, Wheezing, Children, Respiratory health, Indoor Air Quality

## Abstract

**Background:**

Concern is increasing about of the role of poor indoor air quality in respiratory disorders especially in children. While the deleterious respiratory health impact of some volatile organic compounds (VOCs) in the home environment has been suggested, daycares have never been investigated. This study aims to investigate the association between VOCs concentrations in daycare facilities and children’s respiratory health.

**Methods:**

This cross-sectional study included 532 children (mean age: 22.3 months; 47.4% female) of the French CRESPI cohort (100 daycares, 2019–2022). VOC/aldehyde (*n* = 54) concentrations were measured on a one-day active sampling in daycares. They were studied individually in 4 categories based on quartiles and grouped in factors using principal component analysis. Wheezing outcomes - ever wheeze, recurrent wheeze (≥ 3 times), and ever wheeze with inhaled corticosteroid (ICS) use - were evaluated by parental questionnaire. Associations between daycare VOC concentrations/ factors and wheezing outcomes were analyzed with Generalized Estimating Equations to account for a possible daycare effect, and adjusted for child age, parental smoking status and education level.

**Results:**

After correction for multiple testing, higher concentrations of methylisobutylketone (Odds-Ratio for Q4 vs. Q1, 95%CI: 2.40, 1.46–3.92; p-trend < 0.001, FDR-adjusted *p* = 0.02) and 1-methoxy-2-propylacetate (2.58, 1.54–4.33; p-trend < 0.001, FDR-adjusted *p* = 0.02) were associated with recurrent wheeze. Associations were further suggested for 1,2,4-trimethylbenzene (2.70, 1.34–5.45; p-trend = 0.01) and decamethylcyclopentasiloxane (2.27, 1.09–4.71; p-trend = 0.02) but were not significant after correction for multiple testing. Similar findings were observed for all wheezing outcomes. Using factor analysis, three identified factors were associated with wheezing outcomes, including a ‘care/cleaning-products’ factor associated with ever wheeze with ICS (OR: 1.22, 1.00-1.47).

**Conclusions:**

Daycare exposure to specific VOCs was associated with wheeze in children. Their identification and control in daycares may reduce potential health risks.

**Trial registration:**

The CRESPI protocol was registered in the clinical trials register - NCT no 04170881.

**Supplementary Information:**

The online version contains supplementary material available at 10.1186/s12940-026-01307-6.

## Introduction

Since the early 2000s, there has been an increasing interest in understanding the relations between the built environment and human health. Especially, there is a continuously increasing concern about the role of poor Indoor Air Quality (IAQ) in the development of respiratory disorders like asthma [1, 2]. Asthma is an inflammatory chronic disease characterized by reversible airway obstruction and airway hyper-responsiveness that affects up to 300k people worldwide [3], leading to enormous public health costs [4]. In France, asthma affects up to 11% of children [5]. Asthma diagnosis is difficult before the age of 5–6 years, but wheezing symptoms in the first years of life, especially when recurrent, are a strong predictor of asthma development [6]. Improvement in asthma therapies over the last 15 years helped to attain good asthma control in many patients [3], but there is currently no cure for asthma and primary prevention remains key. While several genetic and environmental determinants have been identified, the prevalence of asthma, which has greatly increased since the second half of the 20th century, remains poorly explained [7]. Nonetheless, early life, when the immune system is under development, has been identified as a critical period to explain wheezing symptoms and the origins of asthma [8]. Exposure to environmental factors, where the air quality has a significant role, can contribute to the onset of wheezing symptoms, and young children are considered more vulnerable to the effects of poor air quality.

Although conclusive proof is still lacking, epidemiological studies indicate that some volatile organic compound (VOC) exposures in the indoor environment may negatively impact respiratory health, especially in children. Recent reviews emphasized the ongoing global burden of early-life VOC exposure on children’s respiratory health [9] and highlighted the association of VOCs from household products with respiratory symptoms, particularly in poorly ventilated spaces [10]. Further findings from an extensive review of concentration–response relationships provide an essential foundation for estimating the burden of disease associated with indoor VOCs, especially between asthma and specific compounds like benzene and toluene [11]. However, findings are mixed; in a systematic review, Nurmatov et al. found weak evidence linking VOC exposure with asthma, though some studies reported formaldehyde and specific aromatic VOCs like benzene and toluene, as potential asthma triggers [12]. More recently, an extended review concluded that exposure to formaldehyde may be associated with a higher risk of new onset of wheeze, and that exposure to VOCs in general may be associated with new-onset asthma, persistent wheeze, asthma symptoms and poor lung function [13], although the level of evidence was rated as low.

Most studies on VOC exposures and respiratory health have focused on the domestic or school environment, and on a limited number of specific VOCs and aldehydes (e.g., formaldehyde, benzene, toluene, xylenes) [14–17]. There is a notable lack of studies that comprehensively integrate concurrent respiratory health assessments in children and VOC measurements within daycares. The issue of IAQ in daycares has gained increasing attention over the past decades. According to Eurostat, almost one-third of children (32.3%) in the EU are enrolled in formal childcare (such as daycares or preschool) for at least one hour per week, 60% of them for more than 30 h per week [18]. Young children are increasingly cared for out-of-home in daycares, kindergartens or pre-primary schools, rather than by parents or relatives at home [19]. More caution should be given to the daycare environments where the IAQ is different from primary or higher schools [20–22].

For the first time, the present study aims to investigate the association between concentrations of 67 indoor air pollutants (VOCs and aldehydes) in daycares and child wheeze, considering both single and multi-pollutant approaches.

## Materials and methods

### Study design

The present cross-sectional analysis was performed as a part of the CRESPI epidemiological study (santé RESPIratoire des enfants en Crèche, https://crespi.vjf.inserm.fr), conducted between November 2019 and early February 2022. Details of the study protocol have been described previously [23]. Briefly, the targeted population was children attending daycares—aged from 3 months to less than 4 years old. Daycares from a random sample of 400 daycares in the Paris Metropolitan area, France, were invited to participate until at least 100 acceptances were reached. Ultimately, 108 daycares accepted to participate (participation rate 55%). Parents of children were initially invited through the daycare manager, who sent invitations to all registered families (estimated total of 5,790 families); 922 families sent in a positive reply coupon indicating their consent for participation and were sent a standardized questionnaire. A total of 551 questionnaires were completed by parents (60% of those who sent positive reply coupons, and estimated overall response rate of 9.5%), as previously described [23, 24].

The CRESPI study, sponsored by the French National Institute for Health and Medical Research (Inserm, references C18-05; ID RCB n° 2018-A02657-48), has been approved by the French ethics committee “Comité de protection des personnes” (CPP Sud - Est I n°2019-38; May 2019) and the French Data Protection Authority “Commission Nationale de l’Informatique et des Libertés” (CNIL n°919185; October 2019). The CRESPI protocol was registered in the clinical trials register (NCT n° 04170881). At least one parent or guardian for each child participating in the study signed a written consent form. All methods were conducted in accordance with ethical standards and relevant guidelines and regulations.

### Parental questionnaire and assessment of children’s respiratory health

The parental standardized questionnaire collected information about the family (e.g., education level, working status, medical history, smoking status, number of children, housing characteristics) and the child’s respiratory health. The main outcome of interest was child wheeze at inclusion, defined by the following three outcomes, as previously described [24]: (i) ever wheeze since birth, if parents indicated that the child experienced wheezing in the chest at any point; (ii) recurrent wheeze, defined by at least 3 episodes of wheezing since birth; (iii) Wheeze with inhaled corticosteroid (ICS) defined by at least one episode of wheezing, along with parental report of ICS treatment.

### Indoor air quality measurements in daycares

One technician was in charge of the one-day measurements in daycares. Aldehydes were sampled actively at an airflow rate of 300 ml/min on a DNPH cartridges for 6 h with a Gilair Plus pump (Gilian). VOCs were sampled actively for 6 h too, at an airflow rate of 20 ml/min on Tenax 60/80 tubes with a Pocket pump (SKC). The flow rates were checked before and after sampling with a TSI 4146 flowmeter (TSI). Aldehyde samples were sent to the laboratory in refrigerated packages (4 °C) immediately after sampling, while VOCs samples were sent simultaneously at ambient temperature.

Aldehydes were analyzed through high-performance liquid chromatography coupled with UV detection (HPLC-UV) and VOCs were analyzed through gas chromatography coupled with mass spectrometry (GC/MS). The target al.dehydes were formaldehyde, acetaldehyde and benzaldehyde sampled on the DNPH cartridge, and hexaldehyde and nonanal sampled on the Tenax tube. VOCs included benzene, toluene, ethylbenzene, xylenes, styrene, decane, undecane, 2-ethylhexanol, limonene, alpha-pinene, linalool, and siloxanes. A total of 67 compounds were targeted. More details are provided elsewhere [23].

Along with VOC and aldehyde sampling, indoor temperature and relative humidity were measured continuously on a 10-minute time step. In parallel with the measurements, the technician completed a questionnaire describing the characteristics of the building and the instrumented room (type of ventilation, presence of mold, etc.).

### Statistical analysis

#### Indoor concentrations

Descriptive statistics were performed for each compound. Concentrations below the limit of detection (LOD, see Table 1) were replaced by half of the limit of detection (LOD/2). The 13 compounds that were detected in < = 10% of the daycares were excluded (see Table S1), resulting in 54 studied VOCs (*n* = 49) and aldehydes (*n* = 5). Before the analysis, all concentrations were transformed in 4 categories based on quartiles [14, 25–27]. The choice of categorizing the VOCs was justified by the high number of values below the LOD or high concentrations for some of them. Also, the quartile categories allowed to assess associations both continuously (quartiles increase) and categorically to investigate the dose response effect from each pollutant exposure (Q2, Q3, Q4 vs. Q1 as the reference). For pollutants with more than half of the concentrations below the LOD (resulting in identical Q1 and median values), exposure was categorized in only 3 groups as “high” (Q4) / “low” (Q3) /non detect (Q1-median) [28].

#### Multi-pollutant factor identification

Multivariate exploratory analysis was carried out to identify groups of VOCs and aldehydes which may share common sources. To achieve that, Principal Component Analysis (PCA) was selected [16]. Before applying the PCA, the data were tested for outlier detection using the Grubb’s test [29]. The identified outliers were replaced by the new maximum value of each compound. Then, in order to approach the normal distribution, the Box Cox transformation was applied [30]. The transformed data were used as input for the PCA analysis applying varimax rotation using R (Psych package). The selection of the proper number of factors was based on the scree plot and the max total explained variance.

#### Potential confounding factors

Child age, parental current smoking status, and highest parental educational attainment, obtained from parental standardized questionnaires, were chosen a priori in accordance to the literature to be included in all adjusted models [24]. Age was treated as a continuous variable (in months), while the other variables were categorical. Parental smoking status was classified as present if at least one parent reported being a current smoker. Parental educational attainment was based on the highest level achieved by either parent and divided into three categories: two years of further study after high school or less, high school and three to four years of further study, and high school and five or more years of study.

#### Associations between pollutant concentrations and wheezing outcomes

Associations between indoor concentrations and wheezing outcomes were evaluated by logistic regressions with Generalized Estimating Equations (GEE, with exchangeable correlation structure), used to take into account a potential center (daycare) effect. We analyzed adjusted associations between (i) each individual compound and (ii) identified multi-pollutant factors, and wheezing outcomes. All models were adjusted for the same confounders (i.e., age, parental current smoking status and educational attainment). Associations were reported as odds ratios (OR) with a 95% confidence interval (CI) for a quartile range change (individual VOC; Q2, Q3, Q4 *versus* Q1) or for a 1-unit increase in factor score (PCA), with *p* < 0.05 as significance level or suggested associations with *p* < 0.10. All tests were 2-sided. For analyses of individual VOC, p-values were also corrected for multiple testing using the False Discovery Rate (FDR) approach using the Benjamini-Hochberg procedure considering associations statistically significant if the FDR-adjusted p-value < 0.05. Statistical analyses were conducted using geepack package in R.

Sensitivity analyses were also performed for checking the robustness and reliability of our results. As a sensitivity test to assess the robustness of our findings to the coding of the exposure variables, we additionally analysed VOC concentrations as continuous variables after Box-Cox transformation to account for skewness and values below the detection limit. Results were compared with the categorical (quartile-based) analyses. To assess robustness of findings to adjustment on additional potential confounders, we performed further sensitivity analyses included adjusting for the measured indoor relative humidity, season (summer, spring / winter, autumn) of daycare visit and area level socioeconomic status (area deprivation), including only daycares without visible mold, including daycare visit since March 2020 or later (when circulation of the Covid-19-causing SARS-CoV-2 virus drastically increased in Europe and social measured were enacted), restricting analyses to children attending daycare for ≥ 11 months, or to children for whom the first episode of wheeze occurred after daycare attendance. Stratified analyses were conducted according to the daycare ventilation system (exhaust-only mechanical ventilation systems vs. balanced systems).

## Results

### Participation, characteristics of the study population and wheezing prevalence

**Fig. 1:**
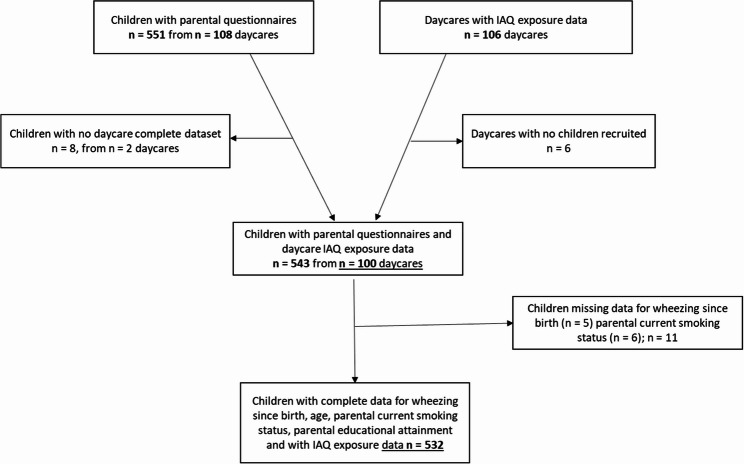
Database flow chart describing the children included in the study of associations between VOC concentrations and wheeze in daycares within the CRESPI cohort

Out of the 551 children included in the CRESPI cohort, 532 had data for wheezing since birth and all selected covariates as well as for air quality data, in 100 daycares (Fig. 1). The age ranged from 3 to 57 months (median 21.7, mean 22.3) and 47.4% of children were female. The prevalence of ever wheeze, recurrent wheeze and wheeze with ICS were 32.1%, 13.3% and 14.8%, respectively. A total of 21.4% of parents reported being current smokers and 79.7% completed at least 5 years of study after high school.

### VOCs and aldehydes in daycares

#### Measured VOC and aldehyde concentrations

The VOC and aldehyde concentrations were differently distributed among the 100 daycares (Table 1). The majority of the compounds were present in above 70% of the daycares. Toluene, acetaldehyde and formaldehyde were detected in all samples (100%), and 2-butanone, benzene, decamethylcyclopentasiloxane (D5) and dodecamethylcyclohexasiloxane (D6) in 99%. The highest median concentrations were measured for acetaldehyde (11.6 µg/m^3^, 95th percentile: 35.7 µg/m^3^), D5 (8.1 µg/m^3^; 95th percentile: 101 µg/m^3^), formaldehyde (7.1 µg/m^3^; 95th percentile: 17.5 µg/m^3^) and 2-butanone (2.7 µg/m^3^; 95th percentile: 68.0 µg/m^3^). The highest mean concentrations were observed for D5 (23.8 µg/m^3^, sd: 42.0 µg/m^3^), acetaldehyde (14.8 µg/m^3^, sd: 10.0 µg/m^3^), limonene (11.9 µg/m^3^, sd: 44.1 µg/m^3^) and 2-butanone (11.2 µg/m^3^, sd: 22.8 µg/m^3^). Spearman’s correlation coefficients between VOCs/aldehydes ranged from 0.01 to 0.87 (Figure S1). The highest correlations were found for o-xylene and p-xylene (0.87, p-value: <0.01), m-xylene and ethylbenzene (0.82, p-value: <0.01), toluene and m-xylene (0.78, p-value: <0.01) and toluene and ethylbenzene (0.77, p-value: <0.01).

**Table 1:** Indoor concentrations (µg/m^3^) of VOCs and aldehydes in 100 randomly selected daycares in the Paris metropolitan area (2019–2022)

Compounds	CAS number	LOD	LOQ^1^	Detection rate %	< LOQ %	25%	median	mean	75%	95%
Acetaldehyde	75-07-0	0.32	0.64	100	0	7.1	11.6	14.8	21.0	35.7
Formaldehyde	50-00-0	0.36	0.64	100	0	5.6	7.1	8.4	10.5	17.5
Toluene	108-88-3	0.14	0.46	100	2	1.3	2.0	2.8	3.2	7.3
Dodecamethylcyclohexasiloxane (D6)	540-97-6	0.14	0.46	99	3	1.1	2.3	3.6	4.2	9.9
2-butanone	78-93-3	0.14	0.46	98	3	1.4	2.7	11.2	7.6	68.0
Benzene	71-43-2	0.14	0.46	98	14	0.6	1.1	1.7	2.5	4.5
Decamethylcyclopentasiloxane (D5)	541-02-6	0.14	0.46	98	3	2.8	8.1	23.8	21.0	101.2
Hexamethylcyclotrisiloxane (D3)	541-05-9	0.14	0.46	97	5	1.5	2.7	4.7	5.7	15.7
Phenol	108-95-2	0.14	0.46	92	10	0.8	1.8	2.5	3.7	6.3
m-xylene	108-38-3	0.14	0.46	92	21	0.5	1.0	1.3	1.5	3.6
Nonanal	124-19-6	0.56	1.85	91	21	2.1	3.8	5.3	6.9	11.8
Octamethylcyclotetrasiloxane (D4)	556-67-2	0.14	0.46	90	43	0.3	0.5	0.7	0.9	1.7
Hexadecane	544-76-3	0.14	0.46	89	60	0.2	0.3	0.9	0.6	2.9
2-ethylhexanol	104-76-7	0.14	0.46	88	22	0.6	2.1	5.1	6.2	18.8
Heptane	142-82-5	0.14	0.46	88	49	0.2	0.5	1.2	1.1	3.9
Ethylbenzene	100-41-4	0.14	0.46	87	59	0.3	0.4	0.5	0.6	1.5
Alpha-pinene	80-56-8 + 7785-26-4	0.14	0.46	86	50	0.3	0.5	6.0	0.8	2.0
Limonene	138-86-3	0.14	0.46	86	20	0.6	2.1	11.9	7.3	45.1
o-xylene	95-47-6	0.14	0.46	85	52	0.2	0.5	0.6	0.7	1.5
1,2,4-trimethylbenzene	95-63-6	0.14	0.46	83	62	0.2	0.4	0.4	0.6	1.0
n-undecane	1120-21-4	0.14	0.46	83	30	0.3	0.8	1.3	1.4	4.1
p-xylene	106-42-3	0.14	0.46	82	53	0.2	0.4	0.6	0.7	1.3
Decanal	112-31-2	0.14	0.46	81	26	0.4	1.3	2.7	3.0	6.9
Pentadecane	629-62-9	0.14	0.46	81	67	0.2	0.3	0.4	0.6	1.2
Dodecane	112-40-3	0.14	0.46	77	46	0.2	0.5	0.9	1.0	2.5
Hexane	110-54-3	0.14	0.46	77	44	0.2	0.6	1.0	1.0	3.0
2-pentanone	107-87-9	0.14	0.46	73	62	< LOD	0.3	0.5	0.7	1.6
Nonane	111-84-2	0.14	0.46	73	70	< LOD	0.3	0.5	0.5	1.4
Decane	124-18-5	0.14	0.46	70	55	< LOD	0.3	0.8	0.9	2.6
Eucalyptol	470-82-6	0.14	0.46	70	49	< LOD	0.5	2.5	1.1	5.0
Octane	111-65-9	0.14	0.46	69	57	< LOD	0.4	0.6	0.6	1.6
Benzaldehyde	100-52-7	0.27	0.91	68	59	0.1	0.6	0.9	1.6	2.8
Dihydromyrcenol	18479-58-8	0.14	0.46	68	63	< LOD	0.3	0.6	0.9	2.3
Hexanal	66-25-1	0.14	0.46	68	40	< LOD	0.9	1.2	1.9	3.5
1-methoxy-2-propanol	107-98-2	0.14	0.46	64	50	< LOD	0.5	1.2	1.5	3.7
2-butoxyethanol	111-76-2	0.14	0.46	63	56	< LOD	0.3	0.8	0.8	3.0
6-methyl-5-hepten-2-one	110-93-0	0.14	0.46	62	59	< LOD	0.4	1.4	1.1	3.5
Acetophenone	98-86-2	0.14	0.46	62	62	< LOD	0.3	0.6	0.8	2.1
Methylcyclohexane	108-87-2	0.14	0.46	57	66	< LOD	0.3	1.1	0.9	4.0
Linalool	78-70-6	0.14	0.46	53	60	< LOD	0.1	0.8	0.8	3.6
1-methoxy-2-propylacetate	108-65-6	0.14	0.46	45	77	< LOD	< LOD	0.4	0.4	1.4
1-butoxy-2-propanol	5131-66-8	0.14	0.46	44	67	< LOD	< LOD	3.0	0.9	14.7
Styrene	100-42-5	0.14	0.46	42	92	< LOD	< LOD	0.2	0.2	0.5
MIBK (methylisobutylketone)	108-10-1	0.14	0.46	31	84	< LOD	< LOD	0.8	0.3	2.3
Alpha-terpineol	98-55-5	0.14	0.46	30	85	< LOD	< LOD	0.4	0.2	1.6
Cyclohexanone	108-94-1	0.14	0.46	27	88	< LOD	< LOD	0.2	0.2	0.8
Pentane	109-66-0	0.14	0.46	27	73	< LOD	< LOD	1.5	0.9	6.6
Naphthalene	91-20-3	0.14	0.46	18	94	< LOD	< LOD	0.1	0.1	0.5
2-phenoxyethanol	122-99-6	2.22	7.40	17	97	< LOD	< LOD	1.8	1.1	5.4
Cyclopentane	287-92-3	0.14	0.46	17	88	< LOD	< LOD	1.2	0.1	1.9
dl-carvone	99-49-0	0.14	0.46	15	96	< LOD	< LOD	0.1	0.1	0.3
Tetrachloroethylene	127-18-4	0.14	0.46	14	95	< LOD	< LOD	0.1	0.1	0.3
2-(2-butoxyethoxy)ethanol	112-34-5	2.08	6.94	13	98	< LOD	< LOD	1.9	1.0	3.8
2-(2-ethoxyethoxy)ethanol	111-90-0	2.08	6.94	10	94	< LOD	< LOD	2.1	1.0	8.2

^1^The laboratory has provided the concentrations between LOD and LOQ. Although these values are subject to greater uncertainty, they have been retained for the statistical analysis purposes. LOD and LOQ are expressed in µg/m^3^

#### VOC and aldehyde factor identification

Five factors (scree plot at Figure S2) were retained from the PCA analysis of the indoor air concentrations (Table 2). The total variance explained by the five extracted factors was 50.3%. The first factor (explaining 20% of the total variance) had high positive loadings of ethylbenzene, toluene, xylenes, and 1,2,4-trimethylbenzene. High positive loadings of dihydromyrcenol, naphthalene, eucalyptol, hexadecane, limonene, pentadecane and n-undecane were present in factor 2 (explaining 10% of the total variance). Factor 3 (explaining 8% of the total variance) was characterized by high positive loadings of decanal, phenol, octamethylcyclotetrasiloxane (D4), hexamethylcyclotrisiloxane (D3), nonanal and 2-butoxyethanol. For factor 4 (explaining 6% of the total variance), higher positive loadings were found for methylisobutylketone (MIBK), 1-methoxy-2-propanol and 1-methoxy-2-propylacetate. Finally, compounds with strong loadings such as acetaldehyde, formaldehyde, decamethylcyclopentasiloxane (D5), linalool and undecane contributed in factor 5 (explaining 5% of the total variance).

**Table 2:** PCA analysis factors loadings of VOCs and aldehydes in daycares^1^

VOCs and Aldehydes	Factors				
	1	2	3	4	5
m-xylene	0.889				
o-xylene	0.861				
p-xylene	0.846		0.313		
Toluene	0.821				
Ethylbenzene	0.794				
1,2,4-trimethylbenzene	0.611				
Hexane	0.608				
Octane	0.580				
Alpha-pinene	0.566				
Methylcyclohexane	0.528				
Styrene	0.489				0.392
Tetrachloroethylene	0.408				
Benzene	0.407	0.501	-0.496		
Nonane	0.403			0.575	
2-butoxyethanol	0.378		0.552		
Linalool	0.364				0.558
Dodecane	0.344	0.645	0.350		
Decane	0.315	0.591			
Nonanal	-0.320	0.353	0.585		
Dihydromyrcenol		0.752			
Pentadecane		0.723			
Naphthalene		0.716			
Eucalyptol		0.675			
Hexadecane		0.654			
Limonene		0.639			0.328
2-pentanone		0.586		0.483	
n-undecane		0.568		0.332	
2-butanone		0.519			
Heptane		0.445		0.385	
Pentane		0.437			
Alpha-terpineol		0.389		0.494	
6-methyl-5-hepten-2-one		0.374	0.365	0.356	
1-butoxy-2-propanol		0.335		0.586	
Acetophenone		-0.301	0.445	0.444	
Decanal			0.847		
Phenol			0.667	0.345	
Octamethylcyclotetrasiloxane (D4)			0.628		
Hexamethylcyclotrisiloxane (D3)			0.583		
2-ethylhexanol			0.487		
Hexanal			0.479		0.366
2-phenoxyethanol			0.386		
1-methoxy-2-propylacetate			0.369	0.630	
MIBK (methylisobutylketone)				0.732	
1-methoxy-2-propanol				0.599	
Cyclohexanone				0.531	0.421
Benzaldehyde				-0.358	
Acetaldehyde					0.714
Formaldehyde					0.577
Decamethylcyclopentasiloxane (D5)					0.532
dl-carvone					0.439
2-(2-butoxyethoxy)ethanol					
2-(2-ethoxyethoxy)ethanol					
Cyclopentane					
Dodecamethylcyclohexasiloxane (D6)					
% of variance	20.5	10.5	8.5	6.0	4.8
Cumulative %	20.5	30.9	39.5	45.5	50.3

^1^Factor loadings above 0.3 are shown

Possible sources associated with these profiles were identified according to the literature. Factor 1 is dominated by aromatic hydrocarbons (like benzene derivatives, BTEX: benzene, toluene, ethylbenzene, xylenes) and other VOCs often associated with vehicle emissions, solvents, and industrial products sources like water-based latex paints, solvent-based paints, decorative arts and adhesives [31–33]. It may represent pollution related to outdoor traffic emissions and specific building materials/wall coverings used in the buildings or some cleaning products. Factor 2 consists of ketones, alkanes, and terpenes like eucalyptol and limonene. It may represent emissions from consumer products, cleaning agents, or fragrances. More specific sources could be decorative cosmetics, fine fragrances, shampoos, toilet soaps and other toiletries as well as household cleaners and detergents [34–36]. Factor 3 includes compounds like siloxanes and aldehydes, often found in cleaning products, detergents, additives as well as personal care products and cosmetics, fragrances and flavoring, or as byproducts of indoor air chemistry. It likely represents household products, flooring emissions and indoor chemical reactions [37–39]. Factor 4 contains glycol ethers and ketones, which are commonly used in paints, coatings, and as solvents. This factor may represent emissions from paints, coatings, gums, resins, paints, varnishes, lacquers as well as from printing/writing inks [40–42]. Lastly, factor 5 includes aldehydes (formaldehyde, acetaldehyde) and siloxanes, which are common indoor air pollutants. This factor might represent compounds that don’t fit neatly into the other categories and suggests that it captures more varied sources. Formaldehyde and acetaldehyde are known to be emitted from building materials (flooring, wall coverings) and furniture [43]. D5 is mainly emitted by specific personal care products [44] such as antiperspirants, hair care, skin care, color and other cosmetics, bath and body products, sunscreen and makeup remover.

### Associations between VOC/aldehyde concentrations and child wheeze

#### Individual VOCs/aldehydes and child wheeze

Table 3 presents the associations between the individual VOCs/aldehydes expressing the increase in quartile as continuous and the three wheezing outcomes in children, for the compounds which had significant (p-trend < 0.001, FDR-adjusted *p* < 0.05) or suggested (p-trend < 0.10, before FDR correction) associations for at least one of the studied outcomes (non-significant associations presented in Table S2). After correction for multiple testing, a significant positive association remained for MIBK forever wheeze (p-trend < 0.001, FDR-adjusted *p* = 0.05) and recurrent wheeze (p-trend < 0.001, FDR-adjusted *p* = 0.02), while 1-methoxy-2-propylacetate remained significantly positively associated with recurrent wheeze (p-trend < 0.001, FDR-adjusted *p* = 0.02). Most compounds showed an OR above 1 for different wheezing outcomes, indicating increased likelihood of wheezing associated with increased compound concentration. Some VOCs, such as D5 and MIBK, showed consistent positive associations with all wheezing outcomes. Associations between concentrations of 1,2,4-trimethylbenzene and 1-methoxy-2-propylacetate and all wheezing outcomes were also suggested. For 6-methyl-5-hepten-2-one, cyclohexanone, D4 and alpha-terpineol, high associations with both recurrent wheeze and wheeze with ICS were observed or suggested. Although many compounds have similar ORs across the three outcomes, the differences suggest that some may have varying impacts on ever wheezing, recurrent wheezing, or wheezing that requires inhaled corticosteroids. In the case of benzene and m-xylene, significant inverse associations were identified with at least one wheezing outcome.

**Table 3:** Statistically significant associations observed between some VOC concentrations and wheezing outcomes

Compounds^1^	Ever wheeze			Recurrent wheeze			Ever wheeze with ICS		
	aOR (CI 95%)^2^	*p*-trend^3^	*p*-trend FDR	aOR (CI 95%)^2^	*p*-trend^3^	*p*-trend FDR	aOR (CI 95%)^2^	*p*-trend^3^	*p*-trend FDR
1,2,4-trimethylbenzene	1.13 ( 0.98–1.30 )	0.08	0.52	**1.38 ( 1.10–1.73 )**	0.01*	0.09	1.21 ( 0.98–1.49 )	0.08	0.47
1-methoxy-2-propylacetate	1.19 ( 1.04–1.35 )	0.01*	0.16	**1.37 ( 1.16–1.63 )**	< 0.001*	0.02*	**1.31 ( 1.11–1.53 )**	< 0.001*	0.06
6-methyl-5-hepten-2-one	1.09 ( 0.93–1.27 )	0.29	0.77	1.19 ( 0.98–1.45 )	0.07	0.42	**1.23 ( 1.04–1.45 )**	0.01*	0.18
Acetophenone	1.06 ( 0.93–1.20 )	0.38	0.86	**1.22 ( 1.01–1.46 )**	0.03*	0.34	1.06 ( 0.88–1.26 )	0.55	0.85
Benzene	**0.87 ( 0.75–1.00 )**	0.05*	0.49	0.85 ( 0.71–1.03 )	0.09	0.45	**0.84 ( 0.72–0.98 )**	0.02*	0.22
Cyclohexanone	1.11 ( 0.98–1.27 )	0.11	0.64	**1.19 ( 1.01–1.41 )**	0.04*	0.34	**1.23 ( 1.05–1.44 )**	0.01*	0.18
Decamethylcyclopentasiloxane (D5)	**1.27 ( 1.08–1.49 )**	< 0.001*	0.11	**1.34 ( 1.06–1.70 )**	0.02*	0.21	1.23 ( 0.99–1.52 )	0.06	0.43
Hexamethylcyclotrisiloxane (D3)	**1.22 ( 1.03–1.43 )**	0.02*	0.24	1.13 ( 0.92–1.39 )	0.24	0.75	1.10 ( 0.90–1.35 )	0.33	0.75
Limonene	1.14 ( 0.96–1.34 )	0.12	0.64	**1.27 ( 1.01–1.60 )**	0.04*	0.34	1.11 ( 0.89–1.39 )	0.36	0.77
Methylisobutylketone (MIBK)	**1.22 ( 1.08–1.38 )**	< 0.001*	0.05*	**1.33 ( 1.13–1.57 )**	< 0.001*	0.02*	**1.23 ( 1.06–1.42 )**	0.01*	0.18
Octamethylcyclotetrasiloxane (D4)	1.15 ( 0.99–1.33 )	0.07	0.52	1.20 ( 0.99–1.45 )	0.06	0.39	1.16 ( 0.98–1.37 )	0.10	0.48
alpha-terpineol	**1.15 ( 1.01–1.30 )**	0.04*	0.40	1.17 ( 0.98–1.38 )	0.08	0.42	**1.20 ( 1.03–1.40 )**	0.02*	0.22
m-xylene	0.94 ( 0.81–1.09 )	0.42	0.86	0.96 ( 0.78–1.19 )	0.70	0.88	**0.83 ( 0.69–1.00 )**	0.05*	0.37

* Significance at ≤ 0.05

^1^No significant associations were observed for the other VOCs and for aldehydes (results presented in Table S2). ^2^aOR: adjusted Odds Ratio. aORs are expressed for one quartile increase in VOC concentration. ^3^*p*-values for trend expressing the increase in quartile as continuous

**Fig. 2:**
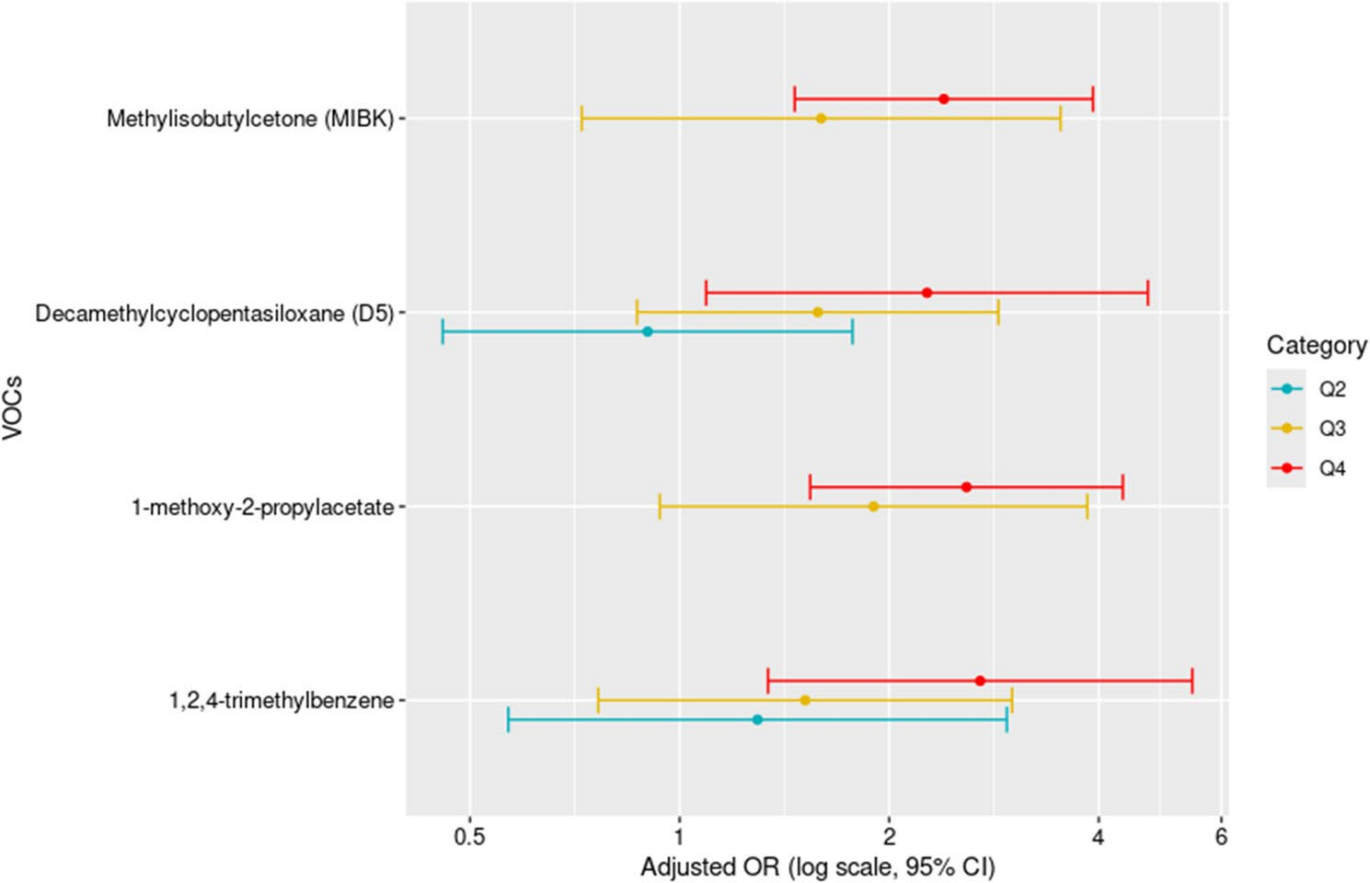
Association between quartiles of selected VOC concentrations with recurrent wheeze. Reference category: concentration < Q1 (or < Q2 when Q1 and Q2 are equal)

Focusing on the compounds with significant positive dose-response associations (Q4, Q3, Q2 vs. Q1), Fig. 2 shows the associations between quartile categories for some VOCs showing positive dose-response with recurrent wheeze. Each category represents a quartile of concentrations (some pollutants have two categories due to the fact that, in more than half of the daycares, the measured concentrations were below the LOD, resulting in the same value for the 25th percentile-Q1 and the median). The reference group (Q1) is not shown, as it serves as the baseline with an OR of 1. Dose-response relationships were observed for the association with recurrent wheeze, with significant associations for the highest (Q4) vs. lowest (Q1) concentrations: the adjusted OR for Q4 vs. Q1 (95% CI) was 2.70 (1.34–5.45) for 1,2,4-trimethylbenzene, 2.58 (1.54–4.33) for 1-methoxy-2-propylacetate, 2.27 (1.09–4.71) for D5 and 2.40 (1.46–3.92) for MIBK. Findings were similar for the two other wheezing outcomes, although associations were not always statistically significant. These results indicate that associations of wheezing in children are increasing and become significant as the indoor concentration of specific compounds increases.

The sensitivity analysis using Box-Cox transformed continuous VOC concentrations (Table S3) showed results comparable to the quartile-based analyses, supporting the robustness of the observed associations. Results of further sensitivity analyses on the four pollutants with positive dose–response relationships (Fig. 2), adjusting for the measured indoor relative humidity, season of daycare visit and area deprivation, remained consistent with the main results (Table S4). Also, the analyses including only those daycares without visible mold showed results consistent with main analyses (Table S4). Stratified analyses, with those for children from daycares with exhaust-only mechanical ventilation systems carried out separately from those with balanced systems, resulted in stronger associations between the four pollutants and wheeze outcomes only for daycares with exhaust-only systems (Table S4). Stratified analyses by duration of daycare attendance (≥ 11 months vs. <11 months) showed generally similar patterns of associations across strata, although some estimates were attenuated and no longer statistically significant in the shorter attendance group. Further sensitivity analyses including daycare visit since March 2020 or later, or to children for whom the first episode of wheeze occurred after daycare attendance, also remained consistent with the results previously described, i.e., 1,2,4-trimethylbenzene, 1-methoxy-2-propylacetate, D5 and MIBK remained associated with at least one wheeze outcome in most analyses (Table S4).

#### Multi-pollutant factors and child wheeze

**Fig. 3:**
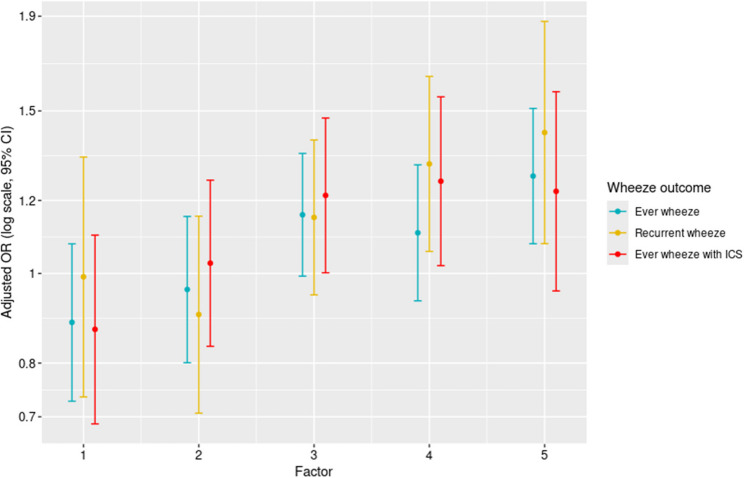
Relationships between VOC and aldehyde factors and wheezing outcome

Figure 3 shows the association between each of the five principal components (factors) from the PCA analysis and different wheezing outcomes in children. Each factor represents a set of related indoor pollutants, as previously reported. For factor 1, which likely represents traffic emissions including 1,2,4-trimethylbenzene, a common constituent of gasoline-fueled vehicle exhaust and specific building materials, and for factor 2, likely associated with consumer products, ORs for all three wheezing outcomes (ever wheeze, ever wheeze with ICS, and recurrent wheeze) were close to 1, with no significant association with any of the wheezing outcomes. For factor 3, which reflects indoor VOCs/aldehydes from cleaning products and personal care products / cosmetics, the ORs for all outcomes ranged from 1.15 to 1.22, with a significant association with ever wheeze with ICS (OR: 1.22; 95%CI 1.00-1.47). For factor 4, related to construction or painting emissions, significant associations were observed for recurrent wheeze (OR: 1.31; 95%CI: 1.06–1.64) and ever wheeze with ICS (OR 1.26; 95%CI: 1.02–1.55). Factor 5, capturing concentrations potentially related to other various compounds emitted by materials and consumer products, showed statistically significant associations with recurrent wheeze (OR: 1.42, 95%CI: 1.08–1.88) and with ever wheeze (OR: 1.28, 95%CI: 1.08–1.51) in children. Sensitivity analyses remained consistent with results previously described, with an attenuation of the statistical significance in some cases or wider confidence intervals due to the restricting of the population (Table S5).

## Discussion

Using indoor air quality data from CRESPI cohort daycares in France, we found an increasing risk of wheezing in children with higher concentrations of compounds such as 1,2,4-trimethylbenzene, 1-methoxy-2-propylacetate, D5 and MIBK. Furthermore, results from the multi-pollutant analysis revealed that groups of VOCs and aldehydes, including those emitted by the use of cleaning and personal care products or from paints, were significantly associated with wheezing symptoms in children.

### VOCs and aldehydes in daycares

Indoor environments include multiple and various sources of VOCs and aldehydes, such as building materials, furniture, paints, glues, flooring, textiles, cleaning supplies and deodorizers. Outdoor emission sources such as traffic, gas stations [45], and small commercial activities such as dry cleaning shops, nail salons and car garages, further influence indoor VOC concentrations. VOCs can adsorb onto surfaces of buildings materials, whereas some materials such as fabrics and other fleecy materials have absorptive properties, acting as pollutant sinks that reemit over time [46, 47]. The latter can be commonly found in daycares (e.g., toys, bedcovers) [48].

The present study is the first to assess a panel of 67 VOCs and aldehydes which were identified and quantified in a sample of 100 daycares. There are very few previous studies assessing IAQ in daycares and most focused on a limited number of VOCs. The median concentrations in our study were below 8 µg/m^3^ except for acetaldehyde. In a recent study in 3 classrooms and 2 hallways in a daycare in Missouri, USA [49], , the mean concentration of 1,2,4-trimethylbenzene was 0.16 µg/m^3^, 0.24 µg/m^3^ for benzaldehyde, 0.25 µg/m^3^ for toluene, 1.29 µg/m^3^ for d-limonene and 0.02 µg/m^3^ for m/p-xylenes, while in our study higher mean values were obtained for these VOCs: 1.3, 0.4, 0.9, 2.8, 11.9 and 1.2 µg/m^3^, respectively. Bayati et al. have also highlighted that 2-ethylhexanol-1, 3-carene and homomenthyl salicylate, emitted from personal care and cleaning products, had the highest indoor concentrations [49]. In another study conducted in 28 French daycares [50], the mean concentration of 1,2,4-trimethylbenzene was 1.5 µg/m^3^, 7.3 µg/m^3^ for toluene and 3.9 µg/m^3^ for m/p-xylenes. Our results are in the same order of magnitude. Benzene, toluene and xylenes (BTX) were the most abundant VOCs in 32 daycares located in Singapore [51], with higher mean concentrations compared to our study.

### VOC and aldehyde concentrations associated with children’s wheezing

Although the current scientific evidence is not yet conclusive, the results of epidemiological studies point toward an adverse effect of VOC exposures on respiratory health in children and adults [9, 10]. Nurmatov and Tagiyeva’s extended review of studies conducted both in children and adults, mainly in households, concluded that the overall evidence for an effect of VOC exposure in asthma development and/or exacerbations was weak mainly because of limitations such as inadequate sampling of VOCs (e.g., short-term sampling), analysis of VOCs that were not related to relevant health outcomes, lack of standardization in assessing asthma, or inadequate adjustment for confounding factors [12]. Also, studies in Australian and Swedish households associated benzene, ethylbenzene, and toluene with asthma in children (Rumchev et al., 2004). However, these previous studies only focused on the home environment and were generally limited in the number of measured pollutants.

In our study, daycare exposure to several specific VOCs and aldehydes was associated with wheezing symptoms in children. In individual pollutant analyses, significant associations were revealed for up to 13 compounds with at least one of the three wheezing outcomes in children, although not all associations were consistent for all three wheezing outcomes. After correction for multiple testing using the false discovery rate, higher concentrations of methylisobutylketone (MIBK) were associated with increased odds of ever wheeze and recurrent wheeze. In addition, higher concentrations of 1-methoxy-2-propylacetate were associated with recurrent wheeze. Several other VOCs showed positive associations with wheezing outcomes before correction for multiple testing, including 1,2,4-trimethylbenzene and decamethylcyclopentasiloxane (D5). However, these associations did not remain statistically significant after FDR correction and should therefore be considered exploratory. For a subset of four compounds (showed indications of dose–response relationships, Fig. 2), associations were observed with all three wheezing outcomes. However, we acknowledge that for certain pollutants, such as MIBK and 1-methoxy-2-propylacetate, the relatively low detection frequencies warrant cautious interpretation, as the estimates rely heavily on values close to or below the LOD. MIBK remained significantly associated with wheezing outcomes in both the continuous and quartile-based analyses, further supporting the robustness of this finding.

### MIBK (methylisobutylketone) 

We found no previous epidemiological study on MIBK and respiratory health. However, specific toxicological studies have reported that clinical tests demonstrated MIBK-induced irritation of the lungs [52–54]. This ketone is a colorless liquid that is used as a solvent for gums, resins, paints, varnishes and lacquers; therefore it is emitted from several building materials in the indoor environment [40].

### 1- methoxy − 2-propylacetate 

We found no previous studies on association between this compound and asthma or wheezing. In our study, this association was observed despite low concentrations in the daycares. This compound can be emitted by products such as paints, lacquers, varnishes, cleaners, ink removers, pesticides, adhesives, dyes for furniture polishes, wood stains, leather and textiles that can be used in indoor environments [55].

### 1,2,4-trimethylbenzene 

One French dwelling study [14] found that a higher 1,2,4-trimethylbenzene concentration in homes was associated with respiratory health in adults. This compound can be produced by flooring emission [33, 40, 56], paints [31] including water-based latex paints and solvent-based paints, and decorative arts; it is also an intermediate in the production of pharmaceutical compounds, perfumes, resins and dyes, and it can be used as a sterilizing agent.

### Decamethylcyclopentasiloxane (D5) 

No epidemiological studies have been found reporting associations between D5 and respiratory health. However, a toxicological study reports that increased incidences of respiratory tract irritation were observed after the inhalation of D5 [44]. In indoor environments, D5 can be emitted by common sources, as it used in cosmetics, such as deodorants, sunblocks, hair sprays and skin care products in bath and body products [57].

Furthermore, in the current study, we found a significant association with child recurrent wheeze for acetophenone and limonene. These compounds are commonly used in fragrances and cosmetic products [40]. They could also be used in deodorizers, air fresheners [34, 36] and cleaning products [56, 58] to give a lemon or orange fragrance. Our findings for limonene are in line with previous studies where an increase in indoor limonene concentration was associated with wheezing and asthma, especially in children [59].

### Decamethylcyclopentasiloxane (D5) 

Alpha-terpineol and octamethylcyclotetrasiloxane (D4), which are commonly used in cosmetic products such as hair and sun care products, skin creams and lotions [37, 60], showed similar associations with child wheeze. In the case of 6-methyl-5-hepten-2-one and cyclohexanone, results showed an association with ever wheeze with ICS. These compounds are usually emitted by personal care products [61] and PVC wallpaper/flooring materials [62], respectively.

Finally, it was observed that benzene and m-xylene were negatively associated (aOR < 1) with wheezing outcomes, which was unexpected. As Norbäck et al. reported, this might be due to residual confounding with some other indoor exposures [63]. Indeed, benzene showed a negative contribution in factor 3 in the PCA analysis, which was correlated with child wheeze. This could occur while other compounds in the same factor (e.g., D4, Acetophenone, D3, decanal, phenol) dominate the exposure profile and have a stronger or more direct effect on health, overshadowing benzene’s potential role. Also, benzene concentrations were negatively correlated with D4 (Spearman = -0.31, p-value: <0.01), acetophenone (-0.39, p-value: <0.01), decanal (-0.36) and phenol (-0.43, p-value: <0.01). In the case of m-xylene, concentrations were negatively corelated with MIBK (-0.32, see figure S1). This significant anticorrelation between -MIBK and m-xylene- as well as between -benzene and acetophenone- could suggest the significant positive/negative association with wheezing outcomes. Alternatively, these associations could be chance findings due to multiple testing.

From the factor analysis, five groups of VOCs and aldehydes have been identified and three of them were associated with wheezing outcomes. First, Factor 3 was associated with wheezing with ICS. This factor is dominated by sources of cleaning and personal care products and cosmetics. These types of products are generally used in daycare environments and can significantly impact indoor air quality. Cleaning products are commonly used for disinfection and sanitation in daycares to maintain hygiene and safety, especially after the COVID-19 pandemic, during which even more extensive usage of these products has been taking place. Prior analyses in the CRESPI cohort showed that higher frequency use of disinfectants and cleaning products in daycares was associated with wheezing in children [24]. Furthermore, personal care products and cosmetics like hand soaps, lotions and sanitizers used by daycare staff or children may contain VOCs used as fragrances or preservatives.

Factor 4 was positively associated with recurrent wheeze episodes and wheeze with ICS. In daycares, compounds characterizing Factor 4 are likely introduced through the use of certain paints, coatings, and other building materials. They can be found in water-based paints, varnishes, and coatings used for wall and furniture maintenance. Also, daycares often repaint or maintain facilities (compounds present in adhesives, glues, and floor finishes) to meet aesthetic and safety standards. Exposure to VOCs and aldehydes from construction or painting activities might contribute to wheezing symptoms in children, especially for those with more severe respiratory issues who require ICS. This factor could reflect exposures to VOCs from renovations or paint, which could exacerbate symptoms. Among pollutants found to be associated individually with wheezing outcomes, 1-methoxy-2-propylacetate and MIBK had high contributions to Factor 4.

Finally, Factor 5 was associated with ever wheeze and recurrent wheeze. This factor represents exposure to compounds emitted from multiple and various sources such as building materials, furnishings, and personal care product use. In daycares, concentrations of formaldehyde and acetaldehyde can be emitted from pressed wood products (e.g., particleboard, plywood, and fiberboard), adhesives, laminates, and insulation materials as well as by wall coverings, floorings (especially vinyl), and paints. Also, D5, which had high loading in Factor 5, can be found in personal care products which are often used by daycare staff or in children’s lotions. This association with ever wheeze in children highlights the influence on respiratory health of chronic exposure to low concentrations of these compounds in daycares. However, factor 5 explained a relatively small proportion of the total variance (4.8%), which may limit its stability and reproducibility. Low-variance factors are more sensitive to sampling variability, and their composition may be less robust compared with factors explaining a larger share of the variance. Therefore, the association observed for this factor should be interpreted with caution.

The cross-sectional design of the study, with exposure assessed after the occurrence of some wheezing outcomes, limits causal interpretation and introduces potential temporal uncertainty. In sensitivity analyses restricted to children whose first wheezing episode occurred after daycare attendance, associations were attenuated for some compounds (1,2,4-trimethylbenzene and D5), while others (MIBK and 1-methoxy-2-propylacetate) remained more robust (Table S4). Stratified analyses by duration of daycare attendance were conducted to partially address temporal uncertainty. While more associations appeared among children with longer attendance, others were attenuated in the shorter-duration group. However, these differences should be interpreted cautiously, as stratification reduced the sample size and thus statistical power, which may explain the lack of statistical significance for certain compounds (e.g., MIBK). Furthermore, the data collection period spanned the COVID-19 pandemic, during which cleaning and disinfection practices in daycares were intensified. This may have influenced indoor VOC concentrations, particularly for compounds related to cleaning products. In addition, changes in daycare attendance patterns during this period may have affected exposure duration and outcome reporting. In sensitivity analyses restricted to the post-March 2020 period, some associations were attenuated, including those for D5 and MIBK. These differences may reflect changes in exposure patterns during the pandemic.

### Strengths and limitations

Our study is the first to investigate associations between individual VOCs/aldehydes and multi-pollutant compound groups with child wheezing outcomes in daycares. The strengths of this study are the relatively large sample of daycares selected in the wider Paris metropolitan area and the inclusion of a broad range of measured VOCs and aldehydes. Furthermore, the multi-pollutant analysis adds a new contribution by taking into consideration the simultaneous presence of numerous compounds in indoor environments. Moreover, the study of early-life wheezing outcomes provides crucial insights into early respiratory symptoms potentially linked to long-term health outcomes. The use of respiratory standardized questionnaires minimizes information bias and allows for more accurate comparisons within the cohort. In the current study, the use of three different outcomes - ever wheeze, recurrent wheeze, and wheeze treated with ICS - provides a more comprehensive assessment of respiratory symptoms and their potential implications for asthma development [24]. Ever wheeze captures any instance of child wheezing, regardless of its frequency or duration and serves as a baseline for exploring associations with indoor pollutants. Recurrent wheeze focuses on the frequency of symptoms, distinguishing between transient, one-time episodes and persistent respiratory issues, and it is more strongly associated with asthma development. Wheeze treated with ICS may reflect the resemblance to asthma, incorporating the judgment of healthcare professionals into the analysis. This layered approach provides a more robust analysis and a comprehensive understanding of child wheezing symptoms which may progress to asthma, and their potential association with the presence of indoor pollutants. Stronger associations were generally observed for more specific wheezing outcome definitions (recurrent wheeze or wheezing with ICS), which are more predictive of asthma development, rather than ever wheeze, which may include children with transient wheezing symptoms.

Some limitations should be noted for this study. Due to the cross-sectional nature of the study, we cannot conclude on causality. The statistical associations observed for individual compounds should be interpreted cautiously, as the possibility that an association is found by chance cannot be excluded. Furthermore, quartile-based associations for chemicals with < 70% detection frequency should be interpreted carefully due to limited variability. We could not formally adjust for exposure to outdoor air pollutants such as NO₂ or PM₂.₅, which are known to be associated with wheezing and may correlate with some VOCs, particularly those related to traffic emissions [64–66]. However, no association was observed between factor 1, which likely represents traffic-related air pollution, and wheezing outcomes in our study. Moreover, although residual confounding by outdoor air pollution therefore cannot be excluded, the use of GEE models accounting for daycare-level clustering partly captures shared environmental characteristics within daycares, which may include broader environmental exposures. Regarding the FDR correction, it was applied separately for each outcome. While this approach is commonly used in observational epidemiological studies [67] also where different outcomes can be treated as separate hypothesis groups [68], it does not fully account for multiplicity across outcomes and may therefore be less conservative. Although the study included a relatively large number (> 500) of children, power was limited in analyses of individual VOCs and aldehydes with correction for multiple testing. However, the analysis of multi-pollutant factors supports an association between several groups of pollutants in daycares and wheezing in children. Another limitation also discussed by previous studies is that the measurements in the daycares were only performed for one day and in one room of the daycare [12]. In daycares, daily activities and major VOC sources, such as cleaning practices, are generally consistent across weekdays. For instance in France, cleaning in nurseries and schools is typically performed daily using relatively standardized procedures [69]. Sampling was conducted under typical weekday conditions, this may support the representativeness of the measurements, although some temporal variability cannot be excluded. VOC concentrations were measured in a single room (main activity room) per daycare, whereas children may use multiple rooms during the day. This may introduce some degree of exposure misclassification, which would bias associations toward the null. Furthermore, the results from the CRESPI cohort may not be generalizable to all populations of children in daycares, whether in France or elsewhere. The participation rate of families was low, at 9.5% [23], but it remains similar to the participation rate in recent, well-known population-based cohorts such as Constances in France (~ 7%) [70]. Furthermore, the prevalence in our sample of parent-reported “ever asthma” (11.7%), “physician-diagnosed asthma” (10.2%) and wheezing (32.1%) in children are similar to prevalence reported in other French populations [5, 71], which does not support strong oversampling of children with asthma or respiratory symptoms. In addition, parents completing the questionnaires were on average highly educated, most with at least five years of further study after high school. Wheezing outcomes were assessed using parental questionnaires, which may introduce information bias. In particular, “ever wheeze since birth” may be subject to recall bias, especially among older children, for whom early-life episodes may be less accurately reported. Such misclassification is likely to be non-differential with respect to exposure and would therefore tend to bias associations toward the null. Finally, a limitation of the data collection for CRESPI was its timing relative to the COVID-19 pandemic, which may introduce bias in the data via modified activities in the daycares (e.g., cleaning and disinfecting) or the voluntary recruitment of participants; however, the sensitivity analysis that was performed showed no influence on the results.

## Conclusions

For the first time, this study investigates the potential impact of exposure to VOCs and aldehydes in daycares on children’s respiratory health. We observed that certain compounds, after correction for multiple testing including MIBK (methylisobutylketone) and 1-methoxy-2-propylacetate significantly associated with wheezing symptoms, while 1,2,4-trimethylbenzene and decamethylcyclopentasiloxane (D5) were suggestively associated with wheezing symptoms, a known precursor to asthma, in children attending daycare. Furthermore, the multi-pollutant factor analysis provided insights into possible combined effects of multiple compounds, suggesting links with three potential sources/categories of pollutants: cleaning products, building materials, and personal care products. While these findings should be interpreted with caution given the cross-sectional design, they nonetheless highlight the importance of further investigation of indoor air quality in daycares. Our results may inform future longitudinal studies and contribute to evidence-based strategies aimed at improving indoor environments and supporting children’s respiratory health in early-life settings.

## Supplementary Information

Supplementary Material 1.

## Data Availability

The authors do not have permission to share data.

## Abbreviations

IAQ: Indoor Air Quality
VOC: Volatile Organic Compound
ICS: Inhaled Corticosteroid
LOD: Limit Of Detection
LOQ: Limit Of Quantification
PCA: Principal Component Analysis
GEE: Generalized Estimating Equations
OR: Odds Ratio
CI: Confidence Interval
FDR: False Discovery Rate
D3: Hexamethylcyclotrisiloxane
D4: Octamethylcyclotetrasiloxane
D5: Decamethylcyclopentasiloxane
D6: Dodecamethylcyclohexasiloxane
MIBK: Methylisobutylketone
