# Supplementary material for: Association between volatile organic compound concentrations in daycares and child wheeze: the French CRESPI study
Source: Environ Health. 2026 May 13;25:58. doi: 10.1186/s12940-026-01307-6 (PMC13340316; doi:10.1186/s12940-026-01307-6)
Supplement: Supplementary file 1 — Supplementary Material 1. [file 12940_2026_1307_MOESM1_ESM.docx]

**Association between Volatile Organic Compound concentrations in daycares and child wheeze. The French CRESPI study.**

**Supplementary material – 8 pages**

Ioannis A. Sakellaris^a^, Corinne Mandin^b^, Franziska Bright^a^, Laurent Orsi^a^, Flore Amat^a,c^, Pierre Bonnet^b^, Valérie Siroux^d^, Nicole Le Moual^a^, Orianne Dumas^a^

^a^ Université Paris-Saclay, UVSQ, Univ. Paris-Sud, Inserm, Équipe d’Épidémiologie Respiratoire Intégrative,

CESP, 94807 Villejuif, France

^b^ Centre Scientifique et Technique du Bâtiment (CSTB), Direction Santé Confort, 84, avenue Jean Jaurès, Marne-la-Vallée (France)

^c^ Service de Pneumologie et d'Allergologie Pédiatrique - CRCM, Hôpital Robert Debré, Université Paris Cité, Paris (France)

^d^ Team of Environmental Epidemiology Applied to the Development and Respiratory Health, Institute for Advanced Biosciences, Inserm U 1209, CNRS UMR 5309, Université Grenoble Alpes, Grenoble (France)

Table S1: Compounds detected in <10% of the daycares

| **Compounds** | **CAS number** | **Detection rate %** |
| --- | --- | --- |
| 1,4-dichlorobenzene | 106-46-7 | 3 |
| 2-butoxy-ethylacetate | 112-07-2 | 0 |
| Geranyl acetate | 105-87-3 | 0 |
| Allyl methyl sulfide | 10152-76-8 | 0 |
| Camphene | 79-92-5 | 6 |
| Citral | 5392-40-5 | 1 |
| Citronellol | 106-22-9 | 0 |
| Dipropylene glycol monomethyl ether | 34590-94-8 | 6 |
| dl-Menthone | 89-80-5 | 7 |
| Geraniol | 106-24-1 | 0 |
| Hydroxycitronellal | 107-75-5 | 0 |
| Terpinolene | 586-62-9 | 3 |
| Trichloroethylene | 79-01-6 | 8 |

Figure S1: Correlation coefficients (Spearman) between indoor VOC concentrations in the daycares


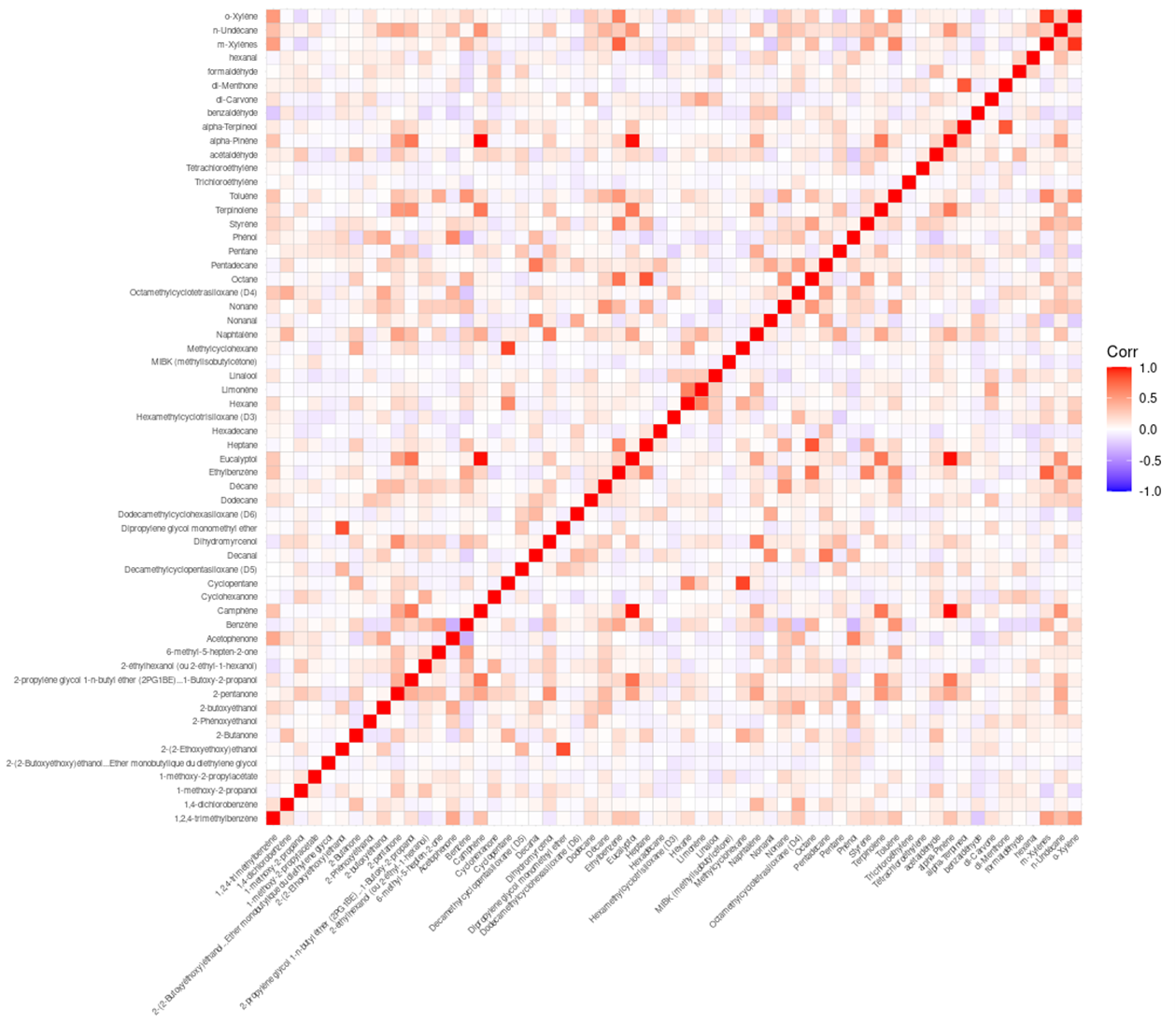


Figure S2: Scree plot of the PCA analyses


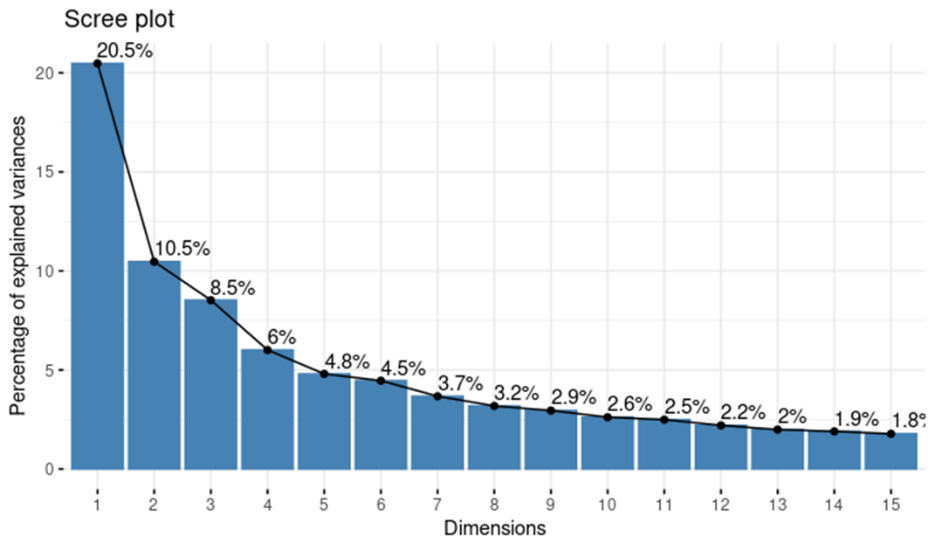


Table S2: Non statistically significant associations between VOC/aldehyde concentrations and wheezing outcomes

|  | **Ever wheeze** | | | **Recurrent wheeze** | | | **Ever wheeze with ICS** | | |
| --- | --- | --- | --- | --- | --- | --- | --- | --- | --- |
| **Pollutant** | **aOR (CI 95%)^1^** | **p-trend** | **p-trend FDR** | **aOR (CI 95%)^1^** | **p-trend** | **p-trend FDR** | **aOR (CI 95%)^1^** | **p-trend** | **p-trend FDR** |
| 1-methoxy-2-propanol | 1.06 ( 0.92 - 1.22 ) | 0.44 | 0.86 | 1.06 ( 0.87 - 1.28 ) | 0.58 | 0.86 | 1.14 ( 0.96 - 1.35 ) | 0.14 | 0.49 |
| 2-(2-butoxyethoxy)ethanol | 0.93 ( 0.78 - 1.11 ) | 0.43 | 0.86 | 0.93 ( 0.74 - 1.16 ) | 0.50 | 0.86 | 1.04 ( 0.86 - 1.26 ) | 0.70 | 0.89 |
| 2-(2-ethoxyethoxy)ethanol | 1.05 ( 0.80 - 1.37 ) | 0.72 | 0.86 | 1.21 ( 0.96 - 1.52 ) | 0.11 | 0.51 | 1.05 ( 0.82 - 1.33 ) | 0.71 | 0.89 |
| 2-butanone | 0.97 ( 0.82 - 1.14) | 0.69 | 0.86 | 0.94 ( 0.75 - 1.17 ) | 0.68 | 0.88 | 1.07 ( 0.86 - 1.34 ) | 0.52 | 0.85 |
| 2-phenoxyethanol | 1.04 ( 0.90 - 1.20 ) | 0.58 | 0.86 | 1.05 ( 0.84 - 1.31 ) | 0.68 | 0.88 | 0.94 ( 0.72 - 1.23 ) | 0.66 | 0.89 |
| 2-butoxyethanol | 1.04 ( 0.89 - 1.21 ) | 0.62 | 0.86 | 1.11 ( 0.89 - 1.39 ) | 0.34 | 0.83 | 1.06 ( 0.86 - 1.31 ) | 0.60 | 0.85 |
| 2-pentanone | 1.03 ( 0.89 - 1.19 ) | 0.67 | 0.86 | 1.06 ( 0.88 - 1.28 ) | 0.52 | 0.86 | 1.17 ( 0.97 - 1.41 ) | 0.11 | 0.48 |
| 1-butoxy-2-propanol | 1.01 ( 0.88 - 1.16 ) | 0.90 | 0.93 | 1.04 ( 0.88 - 1.24 ) | 0.62 | 0.86 | 1.02 ( 0.87 - 1.20 ) | 0.81 | 0.89 |
| 2-ethylhexanol | 1.03 ( 0.88 - 1.20 ) | 0.72 | 0.86 | 0.94 ( 0.77 - 1.15 ) | 0.54 | 0.86 | 1.01 ( 0.84 - 1.22 ) | 0.91 | 0.95 |
| Cyclopentane | 0.95 ( 0.79 - 1.15 ) | 0.61 | 0.86 | 1.05 ( 0.87 - 1.27 ) | 0.62 | 0.86 | 1.00 ( 0.84 - 1.19 ) | 0.97 | 0.99 |
| Decanal | 1.09 ( 0.95 - 1.26 ) | 0.21 | 0.76 | 1.09 ( 0.90 - 1.32 ) | 0.40 | 0.84 | 1.13 ( 0.93 - 1.37 ) | 0.21 | 0.59 |
| Dihydromyrcenol | 0.94 ( 0.81 - 1.10 ) | 0.45 | 0.86 | 0.95 ( 0.78 - 1.16 ) | 0.60 | 0.84 | 0.97 ( 0.81 - 1.17 ) | 0.78 | 0.89 |
| Dodecamethylcyclohexasiloxane (D6) | 1.12 ( 0.96 - 1.31 ) | 0.14 | 0.64 | 1.11 ( 0.90 - 1.36 ) | 0.33 | 0.83 | 1.02 ( 0.86 - 1.22 ) | 0.80 | 0.89 |
| Dodecane | 0.97 ( 0.84 - 1.13 ) | 0.71 | 0.86 | 1.00 ( 0.83 - 1.20 ) | 0.99 | 0.99 | 1.08 ( 0.89 - 1.31 ) | 0.41 | 0.82 |
| Decane | 0.94 ( 0.81 - 1.09 ) | 0.39 | 0.86 | 1.02 ( 0.84 - 1.23 ) | 0.88 | 0.95 | 1.07 ( 0.89 - 1.28 ) | 0.48 | 0.84 |
| Ethylbenzene | 0.96 ( 0.82 - 1.13 ) | 0.64 | 0.86 | 0.96 ( 0.78 - 1.18 ) | 0.70 | 0.88 | 0.87 ( 0.73 - 1.04 ) | 0.12 | 0.48 |
| Eucalyptol | 1.04 ( 0.89 - 1.21 ) | 0.60 | 0.86 | 1.03 ( 0.84 - 1.27 ) | 0.76 | 0.89 | 1.04 ( 0.86 - 1.25 ) | 0.68 | 0.89 |
| Heptane | 0.98 ( 0.83 - 1.15 ) | 0.80 | 0.88 | 0.99 ( 0.79 - 1.24 ) | 0.92 | 0.96 | 1.08 ( 0.86 - 1.35 ) | 0.50 | 0.85 |
| Hexadecane | 0.93 ( 0.81 - 1.07 ) | 0.34 | 0.86 | 0.92 ( 0.77 - 1.10 ) | 0.36 | 0.84 | 0.90 ( 0.75 - 1.08 ) | 0.25 | 0.64 |
| Hexane | 1.02 ( 0.86 - 1.21 ) | 0.80 | 0.88 | 1.14 ( 0.91 - 1.42 ) | 0.25 | 0.75 | 1.17 ( 0.93 - 1.46 ) | 0.17 | 0.54 |
| Linalool | 1.11 ( 0.96 - 1.28 ) | 0.14 | 0.64 | 1.16 ( 0.96 - 1.41 ) | 0.13 | 0.54 | 1.16 ( 0.97 - 1.40 ) | 0.11 | 0.48 |
| Methylcyclohexane | 0.93 ( 0.81 - 1.08 ) | 0.35 | 0.86 | 0.95 ( 0.79 - 1.15 ) | 0.62 | 0.86 | 0.98 ( 0.81 - 1.18 ) | 0.79 | 0.89 |
| Naphthalene | 1.00 ( 0.85 - 1.18 ) | 0.97 | 0.97 | 1.02 ( 0.84 - 1.24 ) | 0.83 | 0.91 | 1.06 ( 0.91 - 1.25 ) | 0.45 | 0.84 |
| Nonanal | 1.04 ( 0.88 - 1.22 ) | 0.65 | 0.86 | 0.95 ( 0.76 - 1.19) | 0.68 | 0.88 | 0.96 ( 0.77 - 1.18 ) | 0.67 | 0.89 |
| Nonane | 0.99 ( 0.86 - 1.14 ) | 0.89 | 0.93 | 1.08 ( 0.90 - 1.30 ) | 0.39 | 0.84 | 1.08 ( 0.90 - 1.29 ) | 0.40 | 0.82 |
| Octane | 1.03 ( 0.89 - 1.19 ) | 0.68 | 0.86 | 0.97 ( 0.80 - 1.18 ) | 0.76 | 0.89 | 1.03 ( 0.86 - 1.24 ) | 0.75 | 0.89 |
| Pentadecane | 0.92 ( 0.80 - 1.05 ) | 0.21 | 0.76 | 0.91 ( 0.75 - 1.10 ) | 0.33 | 0.83 | 0.95 ( 0.78 - 1.15 ) | 0.59 | 0.85 |
| Pentane | 1.08 ( 0.95 - 1.23 ) | 0.23 | 0.77 | 1.03 ( 0.87 - 1.22 ) | 0.72 | 0.88 | 1.09 ( 0.93 - 1.28 ) | 0.29 | 0.71 |
| Phenol | 1.06 ( 0.91 - 1.22 ) | 0.47 | 0.86 | 1.12 ( 0.92 - 1.36 ) | 0.25 | 0.75 | 1.15 ( 0.95 - 1.38 ) | 0.15 | 0.50 |
| Styrene | 1.04 ( 0.90 - 1.20 ) | 0.61 | 0.86 | 1.10 ( 0.91 - 1.34 ) | 0.33 | 0.83 | 1.03 ( 0.86 - 1.23 ) | 0.75 | 0.89 |
| Toluene | 0.92 ( 0.78 - 1.07 ) | 0.27 | 0.77 | 0.94 ( 0.77 - 1.15 ) | 0.55 | 0.86 | 0.89 ( 0.73 - 1.07 ) | 0.22 | 0.59 |
| Tetrachloroethylene | 1.06 ( 0.89 - 1.25 ) | 0.52 | 0.86 | 1.11 ( 0.85 - 1.45 ) | 0.44 | 0.86 | 0.79 ( 0.55 - 1.13 ) | 0.19 | 0.57 |
| Acetaldehyde | 1.02 ( 0.87 - 1.19 ) | 0.79 | 0.88 | 1.09 ( 0.89 - 1.35 ) | 0.40 | 0.84 | 1.11 ( 0.90 - 1.37 ) | 0.33 | 0.75 |
| alpha-pinene | 1.05 ( 0.90 - 1.23 ) | 0.53 | 0.86 | 1.02 ( 0.84 - 1.25 ) | 0.82 | 0.91 | 1.06 ( 0.88 - 1.27 ) | 0.55 | 0.85 |
| Benzaldehyde | 1.01 ( 0.87 - 1.16 ) | 0.93 | 0.95 | 0.87 ( 0.72 - 1.06 ) | 0.17 | 0.67 | 0.98 ( 0.81 - 1.20 ) | 0.87 | 0.92 |
| dl-carvone | 1.12 ( 0.91 - 1.37 ) | 0.28 | 0.77 | 1.08 ( 0.85 - 1.36) | 0.54 | 0.86 | 1.00 ( 0.77 - 1.30 ) | 0.99 | 0.99 |
| Formaldehyde | 1.11 ( 0.95 - 1.31 ) | 0.19 | 0.76 | 1.16 ( 0.92 - 1.46 ) | 0.21 | 0.75 | 1.08 ( 0.88 - 1.32 ) | 0.45 | 0.84 |
| Hexanal | 1.08 ( 0.94 - 1.24 ) | 0.28 | 0.77 | 1.00 ( 0.84 - 1.20 ) | 0.99 | 0.99 | 1.15 ( 0.96 - 1.39 ) | 0.14 | 0.49 |
| n-undecane | 0.98 ( 0.84 - 1.13 ) | 0.74 | 0.86 | 1.01 ( 0.82 - 1.26 ) | 0.89 | 0.95 | 1.08 ( 0.87 - 1.34 ) | 0.48 | 0.84 |
| o-xylene | 0.97 ( 0.83 - 1.13 ) | 0.69 | 0.86 | 1.07 ( 0.85 - 1.35 ) | 0.56 | 0.86 | 0.94 ( 0.76 - 1.16 ) | 0.57 | 0.85 |
| p-xylene | 0.99 ( 0.85 - 1.14 ) | 0.78 | 0.93 | 1.03 ( 0.82 - 1.30 ) | 0.77 | 0.89 | 0.98 ( 0.80 - 1.21 ) | 0.87 | 0.92 |

^1^ aOR: adjusted Odds Ratio. aORs are expressed for one quartile increase in VOC concentration

Table S3: Sensitivity analyses for the associations between VOC continuous Box-Cox transformed concentrations and child wheeze

|  |  | **Ever wheeze** | | | **Recurrent wheeze** | | | **Ever wheeze with ICS** | | | |  |
| --- | --- | --- | --- | --- | --- | --- | --- | --- | --- | --- | --- | --- |
| **Pollutant** | **Detection rate %** | **aOR^1^ (CI 95%)** | **p-value** | **p-value FDR** | **aOR^1^ (CI 95%)** | **p-value** | **p-value FDR** | | **aOR^1^ (CI 95%)** | **p-value** | **p-value FDR** | |
| **Acetaldehyde** | 100 | 1.01 (0.82 - 1.25) | 0.92 | 0.97 | 1.10 (0.83 - 1.47) | 0.51 | 0.81 | | 1.10 (0.82 - 1.47) | 0.52 | 0.78 | |
| **Formaldehyde** | 100 | 1.11 (0.93 - 1.33) | 0.27 | 0.70 | 1.15 (0.87 - 1.52) | 0.33 | 0.75 | | 0.99 (0.78 - 1.26) | 0.96 | 1.00 | |
| **Toluene** | 100 | 0.88 (0.67 - 1.15) | 0.35 | 0.74 | 0.95 (0.63 - 1.45) | 0.83 | 0.92 | | 0.84 (0.58 - 1.20) | 0.33 | 0.77 | |
| **Dodecamethylcyclohexasiloxane (D6)** | 99 | **1.20 (1.00 - 1.45)** | **0.05** | 0.39 | 1.14 (0.86 - 1.51) | 0.37 | 0.75 | | 1.09 (0.85 - 1.39) | 0.49 | 0.78 | |
| **2-butanone** | 98 | 0.95 (0.84 - 1.09) | 0.47 | 0.74 | 0.94 (0.80 - 1.09) | 0.40 | 0.75 | | 1.03 (0.88 - 1.21) | 0.71 | 0.90 | |
| **Benzene** | 98 | **0.86 (0.74 - 1.00)** | **0.05** | 0.39 | **0.82 (0.67 - 1.00)** | **0.05** | 0.29 | | 0.85 (0.72 - 1.01) | 0.06 | 0.25 | |
| **Decamethylcyclopentasiloxane (D5)** | 98 | **1.20 (1.07 - 1.35)** | **0.00** | 0.06 | **1.24 (1.05 - 1.48)** | **0.01** | 0.14 | | **1.21 (1.05 - 1.40)** | **0.01** | 0.16 | |
| **Hexamethylcyclotrisiloxane (D3)** | 97 | **1.19 (1.05 - 1.34)** | **0.00** | 0.09 | 1.09 (0.92 - 1.29) | 0.31 | 0.75 | | **1.17 (1.00 - 1.37)** | **0.05** | 0.23 | |
| **Phenol** | 92 | 1.05 (0.92 - 1.19) | 0.47 | 0.74 | 1.14 (0.96 - 1.36) | 0.14 | 0.53 | | **1.20 (1.01 - 1.42)** | **0.03** | 0.21 | |
| **m-xylene** | 92 | 0.92 (0.77 - 1.11) | 0.40 | 0.74 | 0.95 (0.72 - 1.26) | 0.73 | 0.90 | | 0.87 (0.68 - 1.11) | 0.26 | 0.73 | |
| **Nonanal** | 91 | 1.12 (0.98 - 1.28) | 0.10 | 0.47 | 1.06 (0.88 - 1.28) | 0.52 | 0.81 | | 1.08 (0.91 - 1.30) | 0.38 | 0.78 | |
| **Octamethylcyclotetrasiloxane (D4)** | 90 | 1.19 (0.99 - 1.43) | 0.06 | 0.42 | **1.33 (1.09 - 1.62)** | **0.00** | 0.06 | | **1.27 (1.04 - 1.55)** | **0.02** | 0.20 | |
| **Hexadecane** | 89 | 0.95 (0.84 - 1.08) | 0.45 | 0.74 | 0.99 (0.84 - 1.16) | 0.88 | 0.92 | | 0.95 (0.80 - 1.12) | 0.52 | 0.78 | |
| **2-ethylhexanol** | 88 | 1.00 (0.91 - 1.10) | 0.95 | 0.97 | 0.93 (0.82 - 1.05) | 0.23 | 0.65 | | 0.98 (0.87 - 1.11) | 0.76 | 0.90 | |
| **Heptane** | 88 | 0.98 (0.86 - 1.12) | 0.76 | 0.89 | 1.04 (0.88 - 1.22) | 0.67 | 0.90 | | 1.07 (0.88 - 1.28) | 0.50 | 0.78 | |
| **Ethylbenzene** | 87 | 0.89 (0.73 - 1.10) | 0.28 | 0.70 | 0.88 (0.65 - 1.18) | 0.39 | 0.75 | | 0.78 (0.60 - 1.00) | **0.05** | 0.23 | |
| **Alpha-pinene** | 86 | 0.95 (0.85 - 1.07) | 0.41 | 0.74 | 0.95 (0.81 - 1.12) | 0.54 | 0.81 | | 0.97 (0.84 - 1.12) | 0.69 | 0.90 | |
| **Limonene** | 86 | 1.09 (0.99 - 1.20) | 0.09 | 0.47 | 1.13 (1.00 - 1.27) | **0.05** | 0.29 | | 1.05 (0.94 - 1.18) | 0.38 | 0.78 | |
| **o-xylene** | 85 | 0.88 (0.70 - 1.11) | 0.29 | 0.70 | 0.99 (0.70 - 1.41) | 0.97 | 0.97 | | 0.90 (0.67 - 1.22) | 0.51 | 0.78 | |
| **1,2,4-trimethylbenzene** | 83 | 1.20 (0.92 - 1.58) | 0.18 | 0.62 | **1.79 (1.21 - 2.67)** | **0.00** | 0.06 | | 1.34 (0.91 - 1.97) | 0.14 | 0.44 | |
| **n-undecane** | 83 | 0.97 (0.86 - 1.10) | 0.67 | 0.83 | 1.07 (0.91 - 1.27) | 0.40 | 0.75 | | 1.08 (0.91 - 1.29) | 0.39 | 0.78 | |
| **p-xylene** | 82 | 0.93 (0.77 - 1.12) | 0.42 | 0.74 | 0.96 (0.73 - 1.28) | 0.80 | 0.92 | | 0.96 (0.74 - 1.25) | 0.78 | 0.90 | |
| **Decanal** | 81 | 1.10 (0.99 - 1.22) | 0.07 | 0.43 | 1.11 (0.97 - 1.27) | 0.15 | 0.53 | | **1.16 (1.02 - 1.33)** | **0.03** | 0.20 | |
| **Pentadecane** | 81 | 0.94 (0.81 - 1.10) | 0.45 | 0.74 | 0.96 (0.76 - 1.21) | 0.75 | 0.90 | | 0.98 (0.79 - 1.22) | 0.89 | 0.96 | |
| **Dodecane** | 77 | 0.96 (0.84 - 1.09) | 0.52 | 0.77 | 1.01 (0.85 - 1.20) | 0.93 | 0.95 | | 1.11 (0.92 - 1.34) | 0.27 | 0.73 | |
| **Hexane** | 77 | 1.02 (0.86 - 1.20) | 0.82 | 0.91 | 1.12 (0.94 - 1.34) | 0.20 | 0.64 | | 1.10 (0.92 - 1.33) | 0.30 | 0.76 | |
| **2-pentanone** | 73 | 1.04 (0.89 - 1.22) | 0.62 | 0.83 | 1.08 (0.89 - 1.31) | 0.44 | 0.80 | | 1.17 (0.96 - 1.43) | 0.13 | 0.44 | |
| **Nonane** | 73 | 1.00 (0.89 - 1.13) | 0.98 | 0.98 | 1.11 (0.94 - 1.30) | 0.22 | 0.65 | | 1.07 (0.91 - 1.25) | 0.41 | 0.78 | |
| **Decane** | 70 | 0.96 (0.86 - 1.07) | 0.48 | 0.74 | 1.04 (0.88 - 1.22) | 0.65 | 0.89 | | 1.07 (0.92 - 1.25) | 0.36 | 0.78 | |
| **Eucalyptol** | 70 | 1.03 (0.93 - 1.14) | 0.53 | 0.77 | 1.04 (0.91 - 1.17) | 0.59 | 0.84 | | 1.04 (0.92 - 1.18) | 0.51 | 0.78 | |
| **Octane** | 69 | 1.01 (0.89 - 1.15) | 0.84 | 0.91 | 0.99 (0.83 - 1.18) | 0.89 | 0.92 | | 1.04 (0.88 - 1.23) | 0.66 | 0.90 | |
| **Benzaldehyde** | 68 | 1.02 (0.88 - 1.18) | 0.82 | 0.91 | 0.89 (0.72 - 1.09) | 0.25 | 0.65 | | 0.97 (0.79 - 1.20) | 0.78 | 0.90 | |
| **Dihydromyrcenol** | 68 | 0.95 (0.84 - 1.08) | 0.42 | 0.74 | 0.97 (0.82 - 1.14) | 0.68 | 0.90 | | 0.99 (0.85 - 1.16) | 0.91 | 0.96 | |
| **Hexanal** | 68 | 1.10 (0.97 - 1.24) | 0.12 | 0.48 | 1.02 (0.88 - 1.20) | 0.77 | 0.90 | | **1.17 (1.00 - 1.37)** | **0.05** | 0.23 | |
| **1-methoxy-2-propanol** | 64 | 1.08 (0.96 - 1.21) | 0.18 | 0.62 | 1.09 (0.94 - 1.27) | 0.24 | 0.65 | | **1.15 (1.01 - 1.32)** | **0.04** | 0.21 | |
| **2-butoxyethanol** | 63 | 1.04 (0.92 - 1.16) | 0.55 | 0.78 | 1.11 (0.95 - 1.29) | 0.18 | 0.59 | | 1.05 (0.90 - 1.22) | 0.54 | 0.79 | |
| **6-methyl-5-hepten-2-one** | 62 | 1.05 (0.95 - 1.17) | 0.36 | 0.74 | 1.12 (0.98 - 1.27) | 0.09 | 0.48 | | **1.14 (1.02 - 1.27)** | **0.02** | 0.20 | |
| **Acetophenone** | 62 | 1.04 (0.94 - 1.14) | 0.47 | 0.74 | **1.17 (1.02 - 1.33)** | **0.03** | 0.23 | | 1.05 (0.92 - 1.20) | 0.45 | 0.78 | |
| **Methylcyclohexane** | 57 | 0.95 (0.87 - 1.04) | 0.24 | 0.70 | 0.98 (0.88 - 1.10) | 0.76 | 0.90 | | 0.98 (0.88 - 1.09) | 0.69 | 0.90 | |
| **Linalool** | 53 | 1.07 (0.99 - 1.17) | 0.10 | 0.47 | 1.10 (0.98 - 1.23) | 0.10 | 0.48 | | 1.09 (0.98 - 1.21) | 0.12 | 0.44 | |
| **1-methoxy-2-propylacetate** | 45 | **1.09 (1.02 - 1.17)** | **0.01** | 0.11 | **1.18 (1.08 - 1.29)** | **0.00** | **0.01** | | **1.14 (1.05 - 1.24)** | **0.00** | 0.12 | |
| **1-butoxy-2-propanol** | 44 | 1.01 (0.94 - 1.08) | 0.84 | 0.91 | 1.03 (0.95 - 1.11) | 0.53 | 0.81 | | 1.01 (0.94 - 1.09) | 0.74 | 0.90 | |
| **Styrene** | 42 | 1.01 (0.97 - 1.04) | 0.67 | 0.83 | 1.02 (0.97 - 1.08) | 0.38 | 0.75 | | 1.00 (0.96 - 1.05) | 0.84 | 0.95 | |
| **MIBK (methylisobutylketone)** | 31 | **1.04 (1.02 - 1.07)** | **0.00** | **0.04** | **1.06 (1.03 - 1.10)** | **0.00** | **0.01** | | **1.05 (1.01 - 1.08)** | **0.01** | 0.15 | |
| **Alpha-terpineol** | 30 | **1.02 (1.00 - 1.04)** | **0.05** | 0.39 | 1.02 (1.00 - 1.05) | 0.11 | 0.48 | | **1.03 (1.00 - 1.05)** | **0.03** | 0.20 | |
| **Cyclohexanone** | 27 | 1.01 (1.00 - 1.02) | 0.11 | 0.47 | **1.01 (1.00 - 1.02)** | **0.05** | 0.29 | | **1.01 (1.00 - 1.02)** | **0.01** | 0.16 | |
| **Pentane** | 27 | 1.02 (0.98 - 1.07) | 0.26 | 0.70 | 1.01 (0.95 - 1.06) | 0.85 | 0.92 | | 1.03 (0.98 - 1.08) | 0.31 | 0.76 | |
| **Naphthalene** | 18 | 1.00 (1.00 - 1.01) | 0.94 | 0.97 | 1.00 (0.99 - 1.01) | 0.85 | 0.92 | | 1.00 (1.00 - 1.01) | 0.45 | 0.78 | |
| **2-phenoxyethanol** | 17 | 1.36 (0.39 - 4.71) | 0.63 | 0.83 | 1.39 (0.21 - 9.16) | 0.73 | 0.90 | | 0.56 (0.06 - 5.53) | 0.62 | 0.88 | |
| **Cyclopentane** | 17 | 1.00 (0.99 - 1.01) | 0.68 | 0.83 | 1.00 (0.99 - 1.01) | 0.56 | 0.81 | | 1.00 (0.99 - 1.01) | 1.00 | 1.00 | |
| **dl-carvone** | 15 | 1.00 (1.00 - 1.01) | 0.24 | 0.70 | 1.00 (1.00 - 1.01) | 0.47 | 0.81 | | 1.00 (0.99 - 1.01) | 0.98 | 1.00 | |
| **Tetrachloroethylene** | 14 | 1.00 (1.00 - 1.01) | 0.61 | 0.83 | 1.00 (0.99 - 1.01) | 0.51 | 0.81 | | 0.99 (0.98 - 1.00) | 0.19 | 0.58 | |
| **2-(2-butoxyethoxy)ethanol** | 13 | 0.56 (0.15 - 2.02) | 0.37 | 0.74 | 0.47 (0.10 - 2.12) | 0.33 | 0.75 | | 1.13 (0.30 - 4.29) | 0.86 | 0.95 | |
| **2-(2-ethoxyethoxy)ethanol** | 10 | 1.38 (0.21 - 9.07) | 0.74 | 0.88 | 3.61 (0.70 - 18.54) | 0.12 | 0.51 | | 1.39 (0.26 - 7.50) | 0.71 | 0.90 | |

^1^ ORs are expressed for one unit increase in box-cox transformed concentrations

Table S4: Sensitivity analyses for the associations between VOC concentrations and child wheeze

|  |  | **Ever wheeze** | | | | **Recurrent wheeze** | | | | **Wheeze ever treated with ICS** | | | |
| --- | --- | --- | --- | --- | --- | --- | --- | --- | --- | --- | --- | --- | --- |
|  | **Pollutant** | **n total** | **% Prevalence** | **OR (95% CI)** | **p** | **n total** | **% Prevalence** | **OR (95% CI)** | **p** | **n total** | **% Prevalence** | **OR (95% CI)** | **p** |
| **Adjusted for relative humidity** |  | 524 | 32.4 |  |  | 424 | 16.5 |  |  | 433 | 18.2 |  |  |
|  | 1,2,4-trimethylbenzene |  |  | 1.15 (0.99 - 1.33) | 0.07 |  |  | **1.41 (1.11 - 1.79)** | **<0.01** |  |  | **1.25 (1.01 - 1.54)** | **0.04** |
|  | 1-methoxy-2-propylacetate |  |  | **1.20 (1.04 - 1.38)** | **0.01** |  |  | **1.39 (1.16 - 1.66)** | **<0.01** |  |  | **1.33 (1.13 - 1.56)** | **<0.01** |
|  | D5 |  |  | **1.28 (1.09 - 1.51)** | **<0.01** |  |  | **1.38 (1.08 - 1.76)** | **0.01** |  |  | **1.24 (1.00 - 1.54)** | **0.05** |
|  | MIBK |  |  | **1.23 (1.08 - 1.40)** | **<0.01** |  |  | **1.37 (1.15 - 1.63)** | **<0.01** |  |  | **1.26 (1.09 - 1.45)** | **<0.01** |
| **Adjusted for season** |  | 524 | 32.4 |  |  | 424 | 16.5 |  |  | 433 | 18.2 |  |  |
|  | 1,2,4-trimethylbenzene |  |  | 1.12 (0.97 - 1.30) | 0.12 |  |  | **1.37 (1.08 - 1.73)** | **0.01** |  |  | 1.18 (0.95 - 1.46) | 0.14 |
|  | 1-methoxy-2-propylacetate |  |  | **1.18 (1.03 - 1.34)** | **0.02** |  |  | **1.35 (1.13 - 1.60)** | **<0.01** |  |  | **1.27 (1.08 - 1.50)** | **<0.01** |
|  | D5 |  |  | **1.31 (1.10 - 1.54)** | **<0.01** |  |  | **1.43 (1.11 - 1.85)** | **0.01** |  |  | **1.28 (1.03 - 1.59)** | **0.03** |
|  | MIBK |  |  | **1.24 (1.10 - 1.40)** | **<0.01** |  |  | **1.36 (1.16 - 1.59)** | **<0.01** |  |  | **1.26 (1.09 - 1.47)** | **<0.01** |
| **Adjusted for area deprivation** |  | 529 | 31.9 |  |  | 430 | 16.3 |  |  | 437 | 17.6 |  |  |
|  | 1,2,4-trimethylbenzene |  |  | 1.13 (0.99 - 1.30) | 0.08 |  |  | **1.38 (1.10 - 1.74)** | **0.01** |  |  | 1.20 (0.97 - 1.49) | 0.09 |
|  | 1-methoxy-2-propylacetate |  |  | **1.17 (1.03 - 1.34)** | **0.02** |  |  | **1.35 (1.13 - 1.61)** | **<0.01** |  |  | **1.28 (1.09 - 1.51)** | **<0.01** |
|  | D5 |  |  | **1.24 (1.04 - 1.48)** | **0.02** |  |  | **1.30 (1.00 - 1.69)** | **0.05** |  |  | 1.18 (0.93 - 1.50) | 0.16 |
|  | MIBK |  |  | **1.22 (1.07 - 1.39)** | **<0.01** |  |  | **1.32 (1.11 - 1.58)** | **<0.01** |  |  | **1.24 (1.05 - 1.45)** | **0.01** |
| **Daycares without visible mold** |  | 516 | 32.8 |  |  | 423 | 16.5 |  |  | 428 | 17.5 |  |  |
|  | 1,2,4-trimethylbenzene |  |  | 1.14 (0.99 - 1.31) | 0.08 |  |  | **1.36 (1.09 - 1.71)** | **0.01** |  |  | 1.22 (0.98 - 1.51) | 0.07 |
|  | 1-methoxy-2-propylacetate |  |  | **1.20 (1.05 - 1.37)** | **0.01** |  |  | **1.36 (1.14 - 1.62)** | **<0.01** |  |  | **1.34 (1.14 - 1.57)** | **<0.01** |
|  | D5 |  |  | **1.30 (1.11 - 1.53)** | **<0.01** |  |  | **1.36 (1.07 - 1.73)** | **0.01** |  |  | **1.29 (1.04 - 1.60)** | **0.02** |
|  | MIBK |  |  | **1.24 (1.09 - 1.40)** | **<0.01** |  |  | **1.35 (1.14 - 1.59)** | **<0.01** |  |  | **1.26 (1.09 - 1.46)** | **<0.01** |
| **Daycare visit March 2020 or later** |  | 438 | 30.4 |  |  | 355 | 14.1 |  |  | 362 | 15.7 |  |  |
|  | 1,2,4-trimethylbenzene |  |  | 1.16 (0.99 - 1.34) | 0.06 |  |  | **1.57 (1.21 - 2.03)** | **<0.01** |  |  | 1.22 (0.97 - 1.53) | 0.10 |
|  | 1-methoxy-2-propylacetate |  |  | **1.17 (1.00 - 1.36)** | **0.05** |  |  | **1.37 (1.10 - 1.71)** | **0.01** |  |  | **1.32 (1.08 - 1.60)** | **0.01** |
|  | D5 |  |  | 1.19 (0.99 - 1.44) | 0.06 |  |  | 1.20 (0.91 - 1.59) | 0.20 |  |  | 1.15 (0.89 - 1.50) | 0.29 |
|  | MIBK |  |  | **1.20 (1.03 - 1.39)** | **0.02** |  |  | 1.24 (0.96 - 1.61) | 0.10 |  |  | 1.12 (0.88 - 1.41) | 0.35 |
| **Daycares with an exhaust-only system** |  | 321 | 34.0 |  |  | 258 | 17.8 |  |  | 262 | 19.1 |  |  |
|  | 1,2,4-trimethylbenzene |  |  | 1.09 (0.87 - 1.36) | 0.44 |  |  | **1.42 (1.00 - 2.01)** | **0.05** |  |  | 1.06 (0.76 - 1.48) | 0.74 |
|  | 1-methoxy-2-propylacetate |  |  | **1.34 (1.13 - 1.58)** | **<0.01** |  |  | **1.56 (1.26 - 1.93)** | **<0.01** |  |  | **1.34 (1.10 - 1.63)** | **<0.01** |
|  | D5 |  |  | **1.44 (1.15 - 1.80)** | **<0.01** |  |  | **1.64 (1.19 - 2.26)** | **<0.01** |  |  | **1.46 (1.11 - 1.94)** | **0.01** |
|  | MIBK |  |  | **1.32 (1.13 - 1.54)** | **<0.01** |  |  | **1.42 (1.16 - 1.75)** | **<0.01** |  |  | **1.26 (1.04 - 1.53)** | **0.02** |
| **Daycares with a balanced system (supply and exhaust with heat recovery)** |  | 179 | 30.7 |  |  | 149 | 16.8 |  |  | 150 | 17.3 |  |  |
|  | 1,2,4-trimethylbenzene |  |  | **1.17 (1.02 - 1.35)** | **0.02** |  |  | **1.34 (1.05 - 1.72)** | **0.02** |  |  | 1.20 (0.75 - 1.91) | 0.45 |
|  | 1-methoxy-2-propylacetate |  |  | 1.00 (0.81 - 1.23) | 0.98 |  |  | 1.19 (0.88 - 1.62) | 0.25 |  |  | **1.27 (1.05 - 1.54)** | **0.02** |
|  | D5 |  |  | 1.13 (0.85 - 1.48) | 0.40 |  |  | 1.03 (0.75 - 1.40) | 0.88 |  |  | 0.92 (0.71 - 1.19) | 0.52 |
|  | MIBK |  |  | 1.07 (0.91 - 1.27) | 0.41 |  |  | 1.18 (0.91 - 1.52) | 0.21 |  |  | **1.07 (0.83 - 1.39)** | **0.60** |
| **Children for whom the first episode of wheeze occurred after daycare attendance** |  | 465 | 21.3 |  |  | 398 | 8.0 |  |  | 407 | 10.1 |  |  |
|  | 1,2,4-trimethylbenzene |  |  | 1.03 (0.87 - 1.22) | 0.74 |  |  | 1.03 (0.77 - 1.40) | 0.83 |  |  | 1.05 (0.80 - 1.39) | 0.71 |
|  | 1-methoxy-2-propylacetate |  |  | 1.15 (0.99 - 1.35) | 0.07 |  |  | **1.33 (1.02 - 1.72)** | **0.03** |  |  | **1.31 (1.05 - 1.63)** | **0.02** |
|  | D5 |  |  | 1.14 (0.94 - 1.38) | 0.18 |  |  | 1.15 (0.81 - 1.63) | 0.43 |  |  | 1.13 (0.84 - 1.51) | 0.43 |
|  | MIBK |  |  | **1.26 (1.09 - 1.45)** | **<0.01** |  |  | **1.35 (1.08 - 1.68)** | **<0.01** |  |  | **1.27 (1.03 - 1.56)** | **0.02** |
| **Children attending daycare for ≥ 11 months** |  | 271 | 35.1 |  |  | 221 | 20.4 |  |  | 224 | 21.4 |  |  |
|  | 1,2,4-trimethylbenzene |  |  | 1.12 (0.92 - 1.37) | 0.27 |  |  | 1.26 (0.98 - 1.62) | 0.07 |  |  | 1.15 (0.90 - 1.47) | 0.27 |
|  | 1-methoxy-2-propylacetate |  |  | 1.18 (0.99 - 1.41) | 0.06 |  |  | **1.33 (1.07 - 1.65)** | **0.01** |  |  | **1.33 (1.09 - 1.63)** | **0.01** |
|  | D5 |  |  | 1.18 (0.94 - 1.49) | 0.16 |  |  | 1.22 (0.91 - 1.63) | 0.19 |  |  | 1.31 (0.97 - 1.77) | 0.08 |
|  | MIBK |  |  | **1.25 (1.06 - 1.47)** | **0.01** |  |  | **1.38 (1.12 - 1.69)** | **<0.01** |  |  | **1.32 (1.09 - 1.61)** | **0.01** |
| **Children attending daycare for < 11 months** |  | 269 | 29.7 |  |  | 216 | 12.5 |  |  | 220 | 14.1 |  |  |
|  | 1,2,4-trimethylbenzene |  |  | 1.16 (0.92 - 1.47) | 0.20 |  |  | **1.73 (1.13 - 1.64)** | **0.01** |  |  | 1.33 (0.91 - 1.96) | 0.14 |
|  | 1-methoxy-2-propylacetate |  |  | 1.18 (0.95 - 1.47) | 0.13 |  |  | **1.43 (1.01 - 2.01)** | **0.04** |  |  | 1.19 (0.84 - 1.67) | 0.33 |
|  | D5 |  |  | **1.38 (1.06 - 1.79)** | **0.02** |  |  | 1.48 (0.98 - 2.22) | 0.06 |  |  | 1.03 (0.73 - 1.45) | 0.87 |
|  | MIBK |  |  | 1.18 (0.97 - 1.44) | 0.10 |  |  | 1.27 (0.90 - 1.79) | 0.18 |  |  | 1.08 (0.80 - 1.47) | 0.62 |

Table S5: Sensitivity analyses for the associations between pollutants’ factors and child wheeze

|  |  | **Ever wheeze** | | | | **Recurrent wheeze** | | | | **Wheeze ever treated with ICS** | | | |  |
| --- | --- | --- | --- | --- | --- | --- | --- | --- | --- | --- | --- | --- | --- | --- |
|  | **Factor** | **n total** | **% Prevalence** | **OR (95% CI)** | **p** | **n total** | **% Prevalence** | **OR (95% CI)** | **p** | **n total** | **% Prevalence** | **OR (95% CI)** | **p** | |
| **Adjusted for relative humidity** |  | 524 | 32.4 |  |  | 424 | 16.5 |  |  | 433 | 18.2 |  |  | |
|  | 1 |  |  | 0.88 (0.72 - 1.08) | 0.22 |  |  | 1.01 (0.74 - 1.38) | 0.96 |  |  | 0.89 (0.71 - 1.13) | 0.35 | |
|  | 2 |  |  | 0.95 (0.77 - 1.16) | 0.61 |  |  | 0.87 (0.65 - 1.17) | 0.36 |  |  | 1.01 (0.79 - 1.28) | 0.94 | |
|  | 3 |  |  | **1.23 (1.02 - 1.49)** | **0.03** |  |  | 1.25 (0.97 - 1.63) | 0.09 |  |  | **1.39 (1.10 - 1.74)** | **<0.01** | |
|  | 4 |  |  | 1.11 (0.92 - 1.33) | 0.27 |  |  | **1.35 (1.07 - 1.71)** | **0.01** |  |  | **1.30 (1.05 - 1.60)** | **0.01** | |
|  | 5 |  |  | **1.29 (1.08 - 1.53)** | **<0.01** |  |  | **1.44 (1.08 - 1.92)** | **0.01** |  |  | 1.20 (0.94 - 1.54) | 0.14 | |
| **Adjusted for season** |  | 524 | 32.4 |  |  | 424 | 16.5 |  |  | 433 | 18.2 |  |  | |
|  | 1 |  |  | 0.87 (0.72 - 1.07) | 0.18 |  |  | 0.97 (0.72 - 1.29) | 0.82 |  |  | 0.85 (0.69 - 1.06) | 0.26 | |
|  | 2 |  |  | 0.97 (0.79 - 1.21) | 0.81 |  |  | 0.94 (0.69 - 1.26) | 0.82 |  |  | 1.08 (0.85 - 1.38) | 0.52 | |
|  | 3 |  |  | **1.18 (0.96 - 1.45)** | **0.11** |  |  | 1.07 (0.79 - 1.46) | 0.82 |  |  | 1.15 (0.91- 1.46) | 0.29 | |
|  | 4 |  |  | 1.13 (0.95 - 1.35) | 0.17 |  |  | **1.38 (1.10 - 1.73)** | **0.01** |  |  | **1.35 (1.08 - 1.68)** | **0.04** | |
|  | 5 |  |  | **1.33 (1.13 - 1.57)** | **<0.01** |  |  | **1.59 (1.20 - 2.10)** | **0.01** |  |  | 1.30 (1.02 - 1.65) | 0.08 | |
| **Adjusted for area deprivation** |  | 529 | 31.9 |  |  | 430 | 16.3 |  |  | 437 | 17.6 |  |  | |
|  | 1 |  |  | 0.88 (0.72 - 1.07) | 0.21 |  |  | 0.97 (0.72 - 1.29) | 0.82 |  |  | 0.88 (0.69 - 1.11) | 0.28 | |
|  | 2 |  |  | 0.94 (0.78 - 1.13) | 0.53 |  |  | 0.94 (0.69 - 1.26) | 0.82 |  |  | 0.99 (0.81 - 1.23) | 0.98 | |
|  | 3 |  |  | **1.16 (1.01 - 1.35)** | **0.03** |  |  | 1.07 (0.79 - 1.46) | 0.82 |  |  | **1.21 (0.99- 1.47)** | **0.05** | |
|  | 4 |  |  | 1.11 (0.93 - 1.32) | 0.22 |  |  | **1.38 (1.10 - 1.73)** | **0.01** |  |  | **1.28 (1.03 - 1.58)** | **0.02** | |
|  | 5 |  |  | **1.25 (1.05 - 1.49)** | **0.01** |  |  | **1.59 (1.20 - 2.10)** | **0.01** |  |  | 1.19 (0.92 - 1.54) | 0.17 | |
| **Daycares without visible mold** |  | 516 | 32.8 |  |  | 423 | 16.5 |  |  | 428 | 17.5 |  |  | |
|  | 1 |  |  | 0.90 (0.73 - 1.09) | 0.28 |  |  | 0.99 (0.73 - 1.32) | 0.92 |  |  | 0.89 (0.70 - 1.14) | 0.36 | |
|  | 2 |  |  | 0.96 (0.79 - 1.15) | 0.64 |  |  | 0.87 (0.67 - 1.12) | 0.29 |  |  | 1.02 (0.83 - 1.25) | 0.87 | |
|  | 3 |  |  | **1.14 (0.97 - 1.35)** | **0.11** |  |  | 1.17 (0.95 - 1.43) | 0.12 |  |  | 1.15 (0.95- 1.39) | 0.16 | |
|  | 4 |  |  | 1.11 (0.94 - 1.32) | 0.22 |  |  | **1.31 (1.04 - 1.65)** | **0.01** |  |  | **1.29 (1.04 - 1.60)** | **0.02** | |
|  | 5 |  |  | **1.31 (1.10 - 1.56)** | **<0.01** |  |  | **1.37 (1.03 - 1.82)** | **0.02** |  |  | **1.29 (1.00 - 1.66)** | **0.05** | |
| **Daycare visit March 2020 or later** |  | 438 | 30.4 |  |  | 355 | 14.1 |  |  | 362 | 15.7 |  |  | |
|  | 1 |  |  | 0.97 (0.77 - 1.23) | 0.81 |  |  | 1.35 (0.92 - 1.98) | 0.13 |  |  | 0.98 (0.73 - 1.31) | 0.89 | |
|  | 2 |  |  | 0.96 (0.79 - 1.17) | 0.68 |  |  | 0.93 (0.71 - 1.22) | 0.59 |  |  | 1.03 (0.82 - 1.31) | 0.79 | |
|  | 3 |  |  | 1.15 (0.98 - 1.35) | 0.09 |  |  | 1.15 (0.93 - 1.42) | 0.20 |  |  | **1.29 (1.05- 1.59)** | **0.02** | |
|  | 4 |  |  | 0.94 (0.74 - 1.20) | 0.61 |  |  | 1.22 (0.86 - 1.74) | 0.27 |  |  | 1.17 (0.84 - 1.63) | 0.36 | |
|  | 5 |  |  | 1.21 (0.98 - 1.50) | 0.07 |  |  | 1.27 (0.89 - 1.80) | 0.19 |  |  | 1.13 (0.80 - 1.58) | 0.49 | |
| **Daycares with an exhaust-only system** |  | 321 | 34.0 |  |  | 258 | 17.8 |  |  | 262 | 19.1 |  |  | |
|  | 1 |  |  | 0.79 (0.62 - 1.02) | 0.07 |  |  | 0.92 (0.62 - 1.36) | 0.67 |  |  | **0.72 (0.55 - 0.95)** | **0.02** | |
|  | 2 |  |  | 0.95 (0.75 - 1.20) | 0.65 |  |  | 0.89 (0.66 - 1.20) | 0.45 |  |  | 1.07 (0.81 - 1.42) | 0.61 | |
|  | 3 |  |  | **1.32 (1.07 - 1.64)** | **0.01** |  |  | **1.38 (1.08 - 1.78)** | **0.01** |  |  | **1.36 (1.06- 1.74)** | **0.02** | |
|  | 4 |  |  | 1.20 (0.94 - 1.53) | 0.15 |  |  | **1.49 (1.09 - 2.03)** | **0.01** |  |  | 1.29 (0.98 - 1.69) | 0.07 | |
|  | 5 |  |  | **1.34 (1.00 - 1.79)** | **0.05** |  |  | **1.57 (1.09 - 2.28)** | **0.02** |  |  | 1.36 (0.89 - 2.07) | 0.16 | |
| **Daycares with a balanced system (supply and exhaust with heat recovery)** |  | 179 | 30.7 |  |  | 149 | 16.8 |  |  | 150 | 17.3 |  |  | |
|  | 1 |  |  | 1.21 (0.88 - 1.66) | 0.25 |  |  | 1.20 (0.85 - 1.70) | 0.30 |  |  | 1.21 (0.83 - 1.75) | 0.37 | |
|  | 2 |  |  | 1.07 (0.79 - 1.46) | 0.67 |  |  | 0.97 (0.67 - 1.41) | 0.87 |  |  | 1.08 (0.86 - 1.35) | 0.50 | |
|  | 3 |  |  | 0.89 (0.69 - 1.14) | 0.34 |  |  | 0.89 (0.62 - 1.26) | 0.50 |  |  | 0.97 (0.76- 1.25) | 0.83 | |
|  | 4 |  |  | 0.97 (0.71 - 1.33) | 0.86 |  |  | 1.08 (0.74 - 1.59) | 0.69 |  |  | 1.26 (0.87 - 1.82) | 0.22 | |
|  | 5 |  |  | **1.23 (1.00 - 1.33)** | **0.05** |  |  | 1.30 (0.94 - 1.79) | 0.12 |  |  | 0.94 (0.79 - 1.12) | 0.50 | |
| **Children for whom the first episode of wheeze occurred after daycare attendance** |  | 465 | 21.3 |  |  | 398 | 8.0 |  |  | 407 | 10.1 |  |  | |
|  | 1 |  |  | 0.79 (0.62 - 1.01) | 0.06 |  |  | 0.74 (0.47 - 1.16) | 0.18 |  |  | 0.85 (0.58 - 1.24) | 0.39 | |
|  | 2 |  |  | 0.96 (0.78 - 1.20) | 0.75 |  |  | 1.04 (0.73 - 1.47) | 0.85 |  |  | 1.04 (0.77 - 1.40) | 0.79 | |
|  | 3 |  |  | 1.13 (0.92 - 1.38) | 0.25 |  |  | 1.24 (0.94 - 1.62) | 0.13 |  |  | 1.32 (0.98 - 1.77) | 0.07 | |
|  | 4 |  |  | 1.07 (0.88 - 1.30) | 0.53 |  |  | 1.18 (0.82 - 1.69) | 0.38 |  |  | 1.16 (0.86 - 1.55) | 0.33 | |
|  | 5 |  |  | 1.17 (0.95 - 1.46) | 0.14 |  |  | 1.18 (0.78 - 1.78) | 0.43 |  |  | 1.02 (0.71 - 1.46) | 0.93 | |
| **Children attending daycare for ≥ 11 months** |  | 271 | 35.1 |  |  | 221 | 20.4 |  |  | 224 | 21.4 |  |  | |
|  | 1 |  |  | 0.84 (0.65 - 1.08) | 0.18 |  |  | 0.89 (0.66 - 1.20) | 0.46 |  |  | 0.89 (0.64 - 1.22) | 0.46 | |
|  | 2 |  |  | 0.81 (0.61 - 1.06) | 0.12 |  |  | 0.84 (0.59 - 1.21) | 0.35 |  |  | 0.89 (0.67 - 1.18) | 0.40 | |
|  | 3 |  |  | 1.12 (0.92 - 1.36) | 0.26 |  |  | 1.13 (0.89 - 1.44) | 0.32 |  |  | 1.09 (0.86- 1.38) | 0.46 | |
|  | 4 |  |  | 1.14 (0.90 - 1.45) | 0.29 |  |  | 1.27 (0.95 - 1.69) | 0.11 |  |  | **1.38 (1.05 - 1.81)** | **0.02** | |
|  | 5 |  |  | **1.30 (1.00 - 1.70)** | **0.05** |  |  | **1.62 (1.15 - 2.28)** | **0.01** |  |  | **1.63 (1.13 - 2.36)** | **0.01** | |
| **Children attending daycare for < 11 months** |  | 269 | 29.7 |  |  | 216 | 12.5 |  |  | 220 | 14.1 |  |  | |
|  | 1 |  |  | 0.96 (0.71 - 1.29) | 0.79 |  |  | 1.32 (0.72 - 2.41) | 0.37 |  |  | 0.88 (0.57 - 1.37) | 0.58 | |
|  | 2 |  |  | 1.17 (0.90 - 1.52) | 0.25 |  |  | 1.01 (0.68 - 1.50) | 0.95 |  |  | 1.18 (0.84 - 1.68) | 0.34 | |
|  | 3 |  |  | 1.18 (0.92 - 1.51) | 0.19 |  |  | 1.16 (0.85 - 1.59) | 0.35 |  |  | **1.41 (1.01- 1.97)** | **0.05** | |
|  | 4 |  |  | 1.10 (0.81 - 1.48) | 0.55 |  |  | 1.40 (0.90 - 2.18) | 0.13 |  |  | 1.09 (0.68 - 1.75) | 0.72 | |
|  | 5 |  |  | 1.27 (0.98 - 1.66) | 0.07 |  |  | 1.33 (0.85 - 2.06) | 0.21 |  |  | 0.91 (0.60 - 1.36) | 0.64 | |
